# Harnessing glycofluoroforms for impedimetric biosensing†

**DOI:** 10.1039/d4sc04409f

**Published:** 2024-09-13

**Authors:** Alice R. Hewson, Henry O. Lloyd-Laney, Tessa Keenan, Sarah-Jane Richards, Matthew I. Gibson, Bruno Linclau, Nathalie Signoret, Martin A. Fascione, Alison Parkin

**Affiliations:** a Department of Chemistry, University of York YO10 5DD York UK alison.parkin@york.ac.uk martin.fascione@york.ac.uk; b Department of Chemistry, The University of Manchester M13 9PL UK; c Manchester Institute of Biotechnology, The University of Manchester M1 7DN UK; d Department of Organic and Macromolecular Chemistry, Ghent University Krijgslaan 281-S4 9000 Gent Belgium; e School of Chemistry, University of Southampton Highfield Southampton SO17 1BJ UK; f Hull York Medical School, University of York YO10 5DD York UK

## Abstract

Glycans play a major role in biological cell–cell recognition and signal transduction but have found limited application in biosensors due to glycan/lectin promiscuity; multiple proteins are capable of binding to the same native glycan. Here, site-specific fluorination is used to introduce protein–glycan selectivity, and this is coupled with an electrochemical detection method to generate a novel biosensor platform. 3F-lacto-*N*-biose glycofluoroform is installed onto polymer tethers, which are subsequently immobilised onto gold screen printed electrodes, providing a non-fouling surface. The impedance biosensing platform is shown to selectively bind cancer-associated galectin-3 compared to control glycans and proteins. To improve the analytical capability, Bayesian statistical analysis was deployed in the equivalent circuit fitting of electrochemical impedance spectroscopy data. It is shown that Markov Chain Monte Carlo (MCMC) analysis is a helpful method for visualising experimental irreproducibility, and we apply this as a quality control step.

## Abstract 2

A glycofluoroform biosensor platform is coupled to an electrochemical readout to detect cancer-associated galectin-3. The electrode modification mitigates against non-specific binding while Bayesian analysis monitors experimental reproducibility.

## Graphical abstract

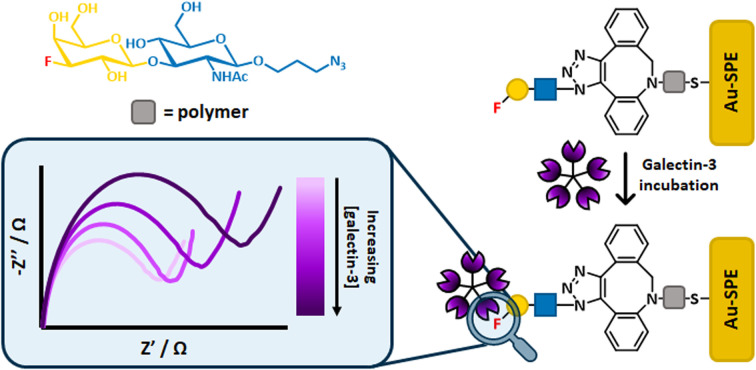

## Introduction

Ideally, a biosensor is a self-contained integral device which is capable of providing specific quantitative analytical information using a bioreceptor.^1^ This paper describes an electrochemical biosensor that utilises an unnatural fluorinated carbohydrate (a glycofluoroform) bioreceptor for the detection of the mammalian carbohydrate binding protein galectin-3. We demonstrate how electrochemical impedance spectroscopy (EIS) can quantify the increase in charge transfer resistance (*R*_ct_) between a gold electrode surface and solution-phase ferricyanide when galectin-3 binds to a surface-confined layer formed from a thiol polymer adorned with a small glycofluoroform bioreceptor that has been designed for selective galectin-3 binding.

Proteins, carbohydrates, nucleic acids and lipids are the four major classes of biological macromolecules. While proteins (often in the form of enzymes and antibodies) and nucleic acids (*e.g.* aptamers) are commonly utilised as bioreceptors in impedimetric biosensors, and lipid layers immobilised on surfaces can be used to capture receptors for sensing,^2–4^ carbohydrates are not widely harnessed as bioreceptors.^5^ This is despite the established major roles that carbohydrate–protein interactions play in a range of biological processes including signalling, cell adhesion, agglutination, protein folding and the immune response.^6–8^ The carbohydrate binding proteins integral to these interactions are known as lectins.^9^ These exhibit reversible and non-covalent carbohydrate binding, both in solution and on surfaces, with substrates of varying complexity.^9,10^ Lectins are sub-classified into large families primarily based on the structure of their carbohydrate recognition domain (CRD), and their carbohydrate binding specificity.^11^ Although lectins can bind oligosaccharides with high specificity, they often bind simpler mono- and disaccharides less discriminately. This innate non-selective small molecule binding,^12^ combined with the fact that multiple lectins can also bind the same carbohydrate, is a significant barrier in the development of carbohydrate receptors for biosensor applications, with only a handful of examples of biosensor platforms in the literature.^13–22^ Such constraints are exemplified by the galectin family – proteins that are prime targets for biosensors because they have been implicated in adhesion and inflammation in several diseases,^23^ with galectin-3 specifically identified as a biomarker for acute and chronic heart failure, liver fibrosis, renal failure, and cancer of the prostate, bladder, thyroid, lung, breast, colon and rectum.^24–27^ The construction of a carbohydrate-based biosensor specific for galectin-3 is challenging because all galectin proteins share a conserved binding site within their CRD.^23^

An emerging solution to the challenge of achieving selective lectin-binding to mono- and disaccharides is the use of glycofluoroforms (GFFs),^28^ unnatural glycomimetics where fluorine atoms typically replace one or more hydroxyl groups in the carbohydrate scaffold. Although the presence of an electronegative fluorine can modulate hydrogen bonding capability and lipophilicity, the conformational effects are often minimal.^29^ This attractive combination of electronic modulation and minimal structural perturbation (a ‘molecular editing’ approach) has previously been applied in the construction of targeted probes and tools for structural biology, rational drug design and medicinal therapeutics,^30,31^ and was recently harnessed in the development of a library of GFFs based on the same carbohydrate scaffold, capable of selective binding to different protein targets.^28,32,33^ Notably, Richards *et al.* showed that in screening experiments using 3FGal-β(1,3)-GlcNAc, a lacto-*N*-biose glycofluoroform, 8-fold selective binding to galectin-3 (*K*_D_ = 6 nM) over galectin-7 could be achieved.^32^

**Fig. 1:**
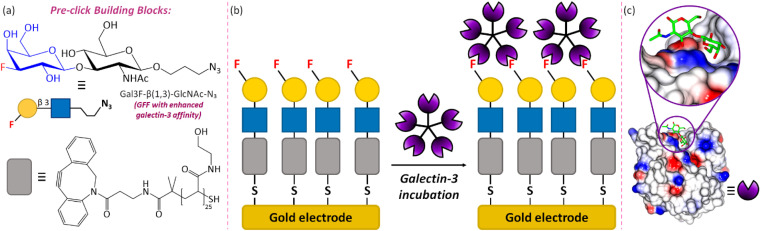
An overview of the GFF-biosensor for galectin-3 detection. (a) The chemical structures and schematic representations of the core building blocks of the sensor: glycofluoroform Gal3F-β(1,3)-GlcNAc-N_3_ and polymeric thiol linker DBCO-(PHEA)_25_-SH. (b) Schematic diagram of the assembled biosensor and the proposed galectin-3 binding interaction. (c) Binding of an analogous native glycoform, LacNAc, on the surface of galectin-3 (PDB: 1KJL).^34^

Herein, we demonstrate how strong, selective binding between 3F-Gal-β(1,3)-GlcNAc and galectin-3 can be harnessed to develop the first GFF electrochemical biosensor (Fig. 1), in which we utilise a thiol terminated polymer–GFF conjugate immobilised on a gold screen-printed electrode (Au-SPE). We note that Fig. 1 depicts a multimeric structure for galectin-3 and a multivalent interaction with the surface in line with the proposed biological function.^35^ The successful detection of galectin-3 by the GFF-biosensor is subsequently demonstrated using EIS, which measures the current–time response to a small amplitude sinusoidal voltage–time oscillation of variable frequency.^36,37^ The data is visualised using both Bode and Nyquist plots to represent changes in impedance, *Z*, the ratio of voltage to current, as a function of frequency. The impedance has a real, *Z*′, and imaginary, *Z*′′, component and the magnitude is denoted |*Z*|. The EIS response is influenced by changes in resistive forces associated with a binding event on the surface of the working electrode. This can be quantitatively determined by equivalent circuit fitting, and such data analysis is significantly more rapid than solving differential equations, as required for the quantitative analysis of voltammetric data. This combination of high surface sensitivity and ease of quantitative analysis makes EIS an attractive detection method for point-of-care diagnostic devices. Indeed, prior studies by other authors have already established that EIS sensors can be directly applied to biofluids with high recovery rates of analytes within clinically relevant ranges.^38–41^ Statistical analytic methods, namely Bayesian inference and Monte Carlo Markov chain, enable us to visualise the confidence and relationship between the modelling parameters used, which highlights the potential of the platform for impedimetric biosensing, and enables critical analysis of the data quality.

## Materials and methods

### Synthesis

The glycofluoroform Gal3F-β(1,3)-GlcNAc-N_3_ was chemoenzymatically synthesised and purified *via* size exclusion chromatography using a modification of a published procedure,^32^ as detailed in the ESI.† The recombinantly expressed enzymes BiGalK and BiGalHexNAcP required were made as described previously.^42,43^

The control sugar, GlcNAc-N_3_, formally 3-azidopropyl 2-acetamido-2-deoxy-β-d-glucopyranoside, was synthesised as described previously.^32^

The polymer, DBCO-(PHEA)_25_-SH, was synthesised as described previously.^32^

### Electrode modification

Gold screen printed electrodes (Au-SPE) produced by BVT were purchased (AC1.R1.R2 2 mm). These comprise a central 2 mm diameter disk working electrode made of gold, a surrounding reference electrode made up of silver covered in silver chloride, and surrounding gold counter electrode printed onto a 7.26 mm × 25.40 mm corundum ceramic chip. All experiments have been conducted in home-built, Perspex gloveboxes with a nitrogen gas supply and Belle purifying recirculation unit (O_2_ ≤ 50 ppm).

Polymer–sugar conjugates were made by reacting the thiol polymer and either the glycofluoroform, Gal3F-β(1,3)-GlcNAc-N_3_, or the control sugar, GlcNAc-N_3_, *via* a strain-promoted azide–alkyne cycloaddition (SPAAC) reaction. Appropriate volumes of polymer (0.32 μmol, 1 equiv.) and sugar (0.64 μmol, 2 equiv.) solutions (both in Milli-Q H_2_O) were added to a 2 mL Eppendorf tube and made up to 450 μL with Milli-Q H_2_O and shaken at room temperature in an anaerobic glovebox environment for 18–24 hours.

Unless otherwise stated, following the SPAAC reaction 450 μL aliquots of the reaction mixture in 2 mL Eppendorf tubes were used to modify two Au-SPEs simultaneously by immersing the SPEs to a depth where the working, counter and reference electrodes were covered. The SPE modification was left to proceed at room temperature in an anaerobic glovebox environment for 18–24 hours. Following Au-SPE modification, the SPAAC reaction aliquots are stored at −20 °C and then thawed for subsequent Au-SPE modifications.

### Protein affinity measurements

Either galectin-3 (recombinant human protein purchased from Abcam, ≥98% purity) or bovine serum albumin (purchased from Sigma-Aldrich) were supplied as freeze-dried powders. The galectin-3 was resuspended in Milli-Q H_2_O to 1 mg mL^−1^ for long term storage (frozen at −20 °C). For protein binding assays using galectin-3, the stock solution was defrosted and appropriate aliquots were diluted in pH 7 aqueous buffer (10 mM HEPES, 150 mM NaCl) to yield the protein concentrations reported in this paper. The bovine serum albumin (BSA) was resuspended in pH 7 aqueous buffer (10 mM HEPES, 150 mM NaCl) to yield the protein concentrations reported in this paper.

To assess the protein binding affinity of a single electrode, if necessary, the electrode was first modified with sugar-polymer. Next, a protein-free initial electrochemical impedance spectroscopy (EIS) measurement was carried out, as described below. The electrode was then rinsed thoroughly to remove all potassium ferricyanide solution before being incubated in the appropriate protein solution, starting at the lowest concentration to be tested within the dataset. The electrode was immersed in approximately 400 μL of protein (sufficient to cover all working electrode surface area) for 30 min (timed using a stopwatch) at room temperature, before being rinsed with Milli-Q H_2_O and measured in a solution of buffered potassium ferricyanide using EIS (*i.e.* electrochemical measurements conducted without protein in solution). Following EIS, the electrode was then rinsed thoroughly with Milli-Q H_2_O before being incubated in the next concentration of protein, followed by another EIS measurement. This two-step, protein incubation followed by EIS measurement, process is repeated for each protein sample to be tested within the dataset, with an incremental increase in concentration each time.

### Electrochemical experimentation

Electrochemical impedance spectroscopy (EIS) measurements were made by applying a 50–100 μL droplet (a sufficient volume to cover all electrodes, dependent on surface tension) of 10 mM potassium ferricyanide in pH 7 aqueous buffer (100 mM sodium phosphate, 233 mM sodium chloride) to the Au-SPE. Experiments were conducted at room temperature in an anaerobic glovebox environment. A PalmSens4 with an EmStatMUX8-R2 multiplexer using PSTrace 5.9 software or an Ivium CompactStat potentiostat with a home built SPE connector using IviumSoft 4.11 were used to perform the EIS measurements, conditions for each experiment are provided in the relevant figure captions.

### Electrochemical analysis

**Fig. 2:**
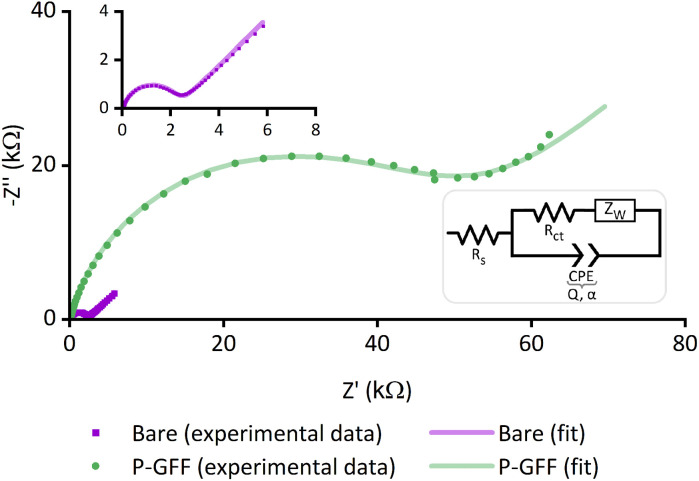
Nyquist plots of experimental data (dots) and the computational fitting to a modified Randles circuit (line) for EIS measurements of a bare gold SPE (purple, *E*_dc_ = 0.160 V *vs.* Ref) and a P–GFF modified gold SPE (green, *E*_dc_ = 0.230 V *vs.* Ref). Inset: magnified view of the Nyquist plot for the bare gold SPE and the modified Randles circuit used in the data analysis. EIS measurements were performed in the presence of 10 mM K_3_[Fe(CN)_6_] in pH 7 aqueous buffer (100 mM sodium phosphate, 233 mM sodium chloride) with the following parameters: *t*_(equilibration)_ = 180 s, *E*_ac_ = 10 mV, *f* = 0.05 Hz–10 kHz.

Each EIS experiment was analysed by fitting the Bode data to a modified Randles circuit (Fig. 2) with two noise parameters using a covariance matrix adaptation evolution strategy (CMA-ES) algorithm to find the ‘best fit’ parameter values.^44^ These were obtained by maximising a Gaussian log-likelihood function (found in the ESI†), and assuming independent and identically distributed noise. The model parameters are as follows: solution resistance, *R*_s_; charge transfer resistance, *R*_ct_; Warburg element, *Z*_w_; double-layer capacitance, modelled by a constant phase element (CPE) made up of *Q* and *α*; and noise parameters for the impedance phase and magnitude, *σ*_1_ and *σ*_2_, respectively. Parameter values were proposed from within hard-coded boundaries for each parameter, as detailed in the ESI.† We fitted to the polar form of the data (Bode plot) because when fitting to the complex form (Nyquist plot) of impedance data, very large complex values (as found at lower frequencies) were over-prioritised relative to the high-frequency values.

To generate posterior distributions for the parameter values, Markov chain Monte Carlo (MCMC) analysis was utilised, using the adaptive covariance MCMC algorithm as implemented in the PINTS repository,^45^ using the same log-likelihood function as maximised by CMA-ES, and an uninformative log-prior using the boundaries for CMA-ES. Three chains were run independently for 10 000 iterations, initialised from the maximum likelihood point determined by CMA-ES. The pooled samples from these chains are plotted as histograms, with the first 3000 samples discarded as “burn-in”. Convergence information can be found in the ESI.†

## Results & discussion

### Electrochemical characterisation of polymer-glycofluoroform modified electrodes

We sought to assemble a galectin-3 EIS biosensor by immobilising our chosen 3F-Gal-β(1,3)-GlcNAc-N_3_ glycofluoroform on a ‘thiophilic’ gold SPE, using a thiol terminating poly(hydroxyethyl acrylamide) polymer as an intermediary platform to maximise display of the ligand on the surface (Fig. 1). A DBCO strained alkyne incorporated within the polymer could be subjected to strain-promoted azide–alkyne cycloaddition (SPAAC)^46^ to efficiently attach the azide containing GFF prior to surface immobilisation, with the polymer intermediary also potentially pacifying the electrode and beneficially minimising non-specific interactions during detection of the analyte.^47^

To prove the formation of gold-thiol bonds between the electrode and GFF-polymer conjugate, we first used EIS to compare the response of ‘bare’ Au-SPEs (*i.e.*, new, from the packet electrodes) to SPEs that have been modified with the SPAAC polymer–glycofluoroform (P–GFF) reaction mixture. Electrochemical measurements carried out in the presence of solution-phase potassium ferricyanide showed the expected response for a solution EIS experiment in the Nyquist plot (Fig. 2),^48^ namely a semi-circle with a linear region at approximately 45°. This indicated that analysis using a modified Randles circuit (Fig. 2, inset) was appropriate, with computational analysis generating a good fit to the experimental data. Notably, a distinct difference can be observed between the bare Au-SPE and the P–GFF modified Au-SPE. The large magnitude of the Nyquist plot for the P–GFF modified Au-SPE relative to a bare Au-SPE indicated that incubation of a Au-SPE in SPAAC P–GFF reaction mixture resulted in gold–thiol bond formation, with the presence of the coating on the gold surface hindering electron transfer between solution ferricyanide and the electrode surface. Numerically, this can be quantified as the change in the electron transfer rate, as discussed later.^49^ Further evidence that incubation of an Au-SPE in P–GFF solution generates a gold–thiol bond is provided by electrochemical ‘stripping’ experiments (Fig. S1†).

Complementary control experiments were also carried out to assess the surface coverage of polymer on the electrode by replacing the glycofluoroform with a ferrocene derivative. A surface-confined ferrocene DCV signal was observed (Fig. S2†) and analysed using a third-order polynomial baseline subtraction to isolate the faradaic current. As detailed in the ESI,† from a simple integration of the total charge passed and assuming a one-electron transfer for each ferrocene molecule, the surface coverage in this experiment was found to be 0.35 pmol which is approximately three-fold lower than the estimated monolayer coverage, 1.01 pmol (Fig. S3†). Since the polymer structure is consistent, and the gold–thiol bond formation chemistry will be unchanged, we also interpret the ferrocene measurements as providing a proxy measurement of the coverage of GFF on the electrode surface. Other control experiments also indicate non-ideal, sub-monolayer formation (*vide infra*).

**Fig. 3:**
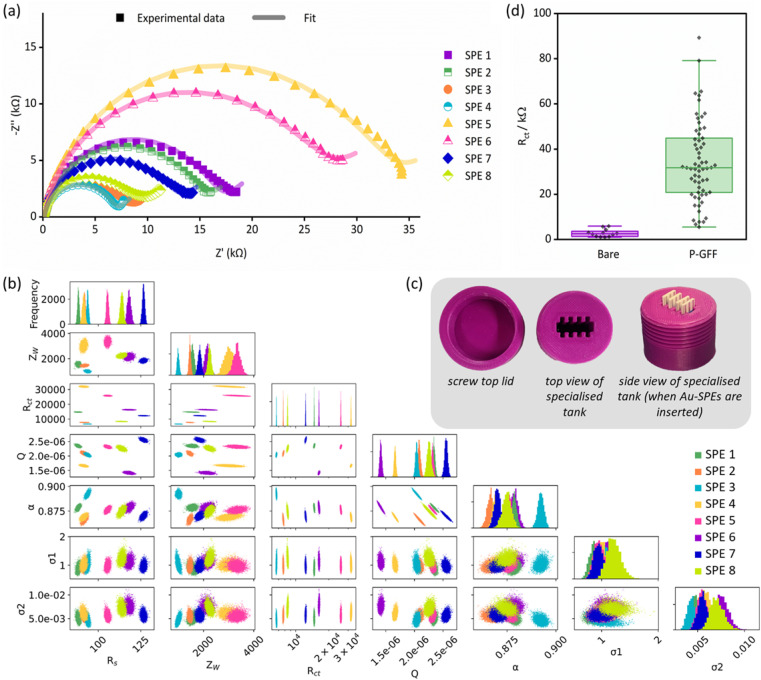
(a) Nyquist plots of experimental data (dots) and the computational fitting to a modified Randles circuit (line) for EIS measurements of eight P–GFF Au-SPEs modified simultaneously. (b) MCMC analysis of the data in (a). The same colour code is used to distinguish between the separate electrodes. The uppermost plots (along the top diagonal) comprise 1-D histograms (*y*-axis is frequency) for each parameter used in equivalent circuit fitting (see *x*-axis labels), showing the distribution of the best-fit values for multiple analysis runs for each individual SPE. The 2-D scatter plots show correlations between parameters. (c) Pictures of the tank used for simultaneous Au-SPE modification. (d) A box and whisker plot showing the variation in the extracted *R*_ct_ parameters for experiments on different ‘bare’, *i.e.* unmodified, Au-SPE (purple, *n* = 12) and different ‘P–GFF’ modified Au-SPEs (green, *n* = 63) following equivalent circuit fitting to the large datasets shown in Fig. S4 and S5.† The limits of the solid box represent the 25th and 75th percentile (20.8 and 44.9 kΩ, respectively), the central horizontal line in the box shows the position of the median (31.7 kΩ) and the upper and lower horizontal “whisker” lines represent the 5th and 95th percentiles (5.5 and 79.1 kΩ, respectively). EIS measurements were performed in the presence of 10 mM K_3_[Fe(CN)_6_] in pH 7 aqueous buffer (100 mM sodium phosphate, 233 mM sodium chloride) with the following parameters: *t*_(equilibration)_ = 300 s, *E*_dc_ = 0.230 V *vs.* Ref, *E*_ac_ = 10 mV, *f* = 0.1 Hz–10 kHz.

EIS experiments comparing bare and P–GFF modified electrodes were repeated extensively (Fig. S4 and S5)† and the Nyquist plots showed substantive variation within the P–GFF dataset that is not present in the bare Au-SPE dataset. This variation could not be reduced by careful control of experimental conditions. Even construction of a specialised tank to enable the simultaneous immersion of eight SPEs in a single aliquot of SPAAC P–GFF reaction mixture (Fig. 3), with agitation to ensure solution homogeneity, identical temperature, concentration, and incubation time, still resulted in a large variation in Nyquist response (Fig. 3(a)). From this data, we concluded electrode modification was inconsistent, which is unsurprising considering extensive pre-modification mechanical and electrochemical polishing is necessary to achieve highly consistent self-assembled Au–thiol monolayer formation on gold disc electrodes.^50^ Such extensive cleaning of the gold surface is not possible when using Au-SPEs.

The Nyquist plots of the dataset comprised of eight simultaneously modified Au-SPEs all exhibit significant variation from one another (Fig. 3(a)) but could all be well modelled by the same modified Randles circuit. To explore if there were any underlying trends in the best fit parameters, we conducted a Markov Chain Monte Carlo (MCMC) analysis to generate posterior fitting distributions that represent the different equivalent circuit model parameters that can give a “best-fit” to the experimental data (Fig. 3(b)).^45^ Within this, we observed the expected correlation between *Q* and *α*, the coupled parameters which together constitute the constant phase element (CPE) used to model double-layer capacitance (diagonal lines in the scatter plot with *Q* on the *x*-axis and *α* on the *y*-axis).^51^ However, the plots showing the frequency distribution for each best fit parameter (the uppermost graphs within Fig. 3(b)) reveal no clear trends in the electrode-to-electrode variation; *i.e.* the ordering of the best fit parameter values with respect to the SPE number (essentially the colour bar code) is different from one parameter frequency distribution to another, meaning there is no clear underlying synergistic trend in the way one parameter changes *versus* another. Alternatively, this lack of pairwise parameter compensation can also be seen by the fact that there is no clear trend in how one parameter value changes relative to another in the 2-D scatter plots where one equivalent circuit model parameter is on the *x*-axis, and another is on the *y*-axis.^45^ Thus, the *R*_ct_ value of a GFF-modified Au-SPE cannot be normalised with respect to another parameter to enable the trivial correction for variation in the dataset.

**Fig. 4:**
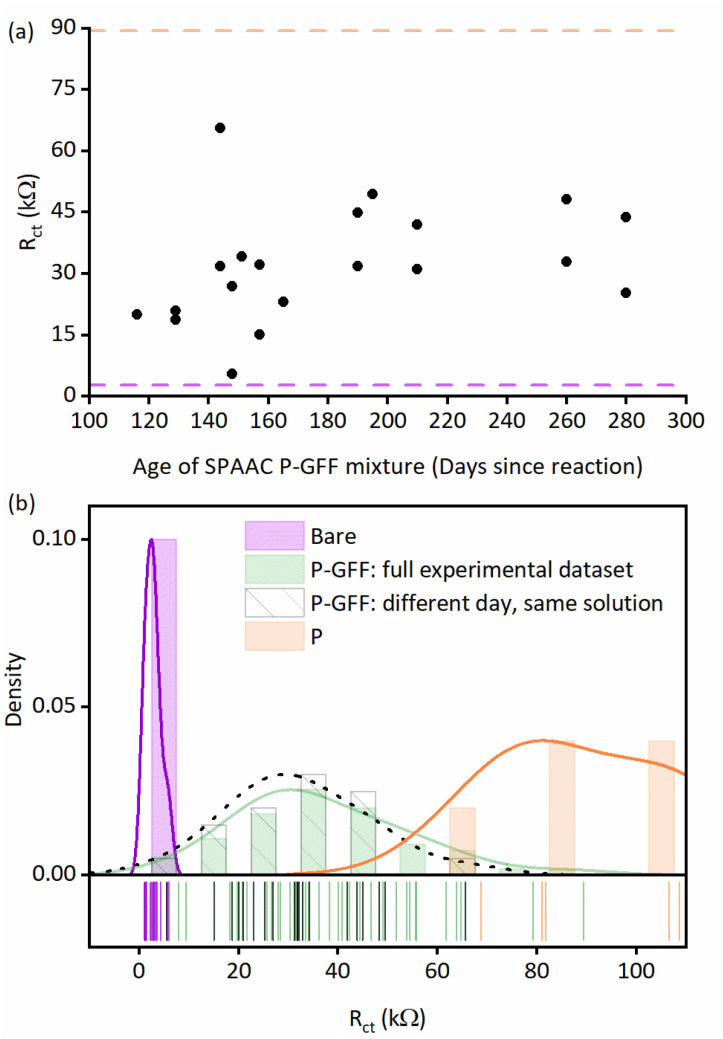
(a) Extracted *R*_ct_ parameters (black dots) for individual P–GFF modified Au-SPEs plotted against the age of the P–GFF conjugate solution showing no correlation in *R*_ct_ over time. The mean *R*_ct_ values (*R̄*_ct_) for both bare (light purple dashed line) and polymer only, “P”, modified (orange dashed line) Au-SPEs are shown for comparison. (b) Distribution plots overlaying the datasets from Fig. 3(d) (purple and green), (a) (grey) and S6† (orange). Lines represent Kernel smooth distributions and a 50% gap is used to separate the vertical bars. The lower portion of the plot is a “rug” representing the point values of the individual measurements.

Since most EIS biosensors are assayed in a manner which correlates change in the charge transfer resistance (*R*_ct_) with target analyte concentration,^2^ the variation in the extracted *R*_ct_ parameters from all the unmodified and P–GFF modified Au-SPEs datasets (Fig. S4 and S5†) was compared (Fig. 3(d)). The 12-fold increase in the average *R*_ct_ for the bare electrodes (2.8 kΩ) *versus* the P–GFF dataset (34.2 kΩ) is equivalent to a 12-fold decrease in the apparent ferricyanide electron transfer rate constant, *k*_app_, of 3.0 μm s^−1^*versus* 0.2 μm s^−1^.^49^ The observed variation between P–GFF modified electrodes was substantive, and this means it is non-trivial to compare repeats across multiple electrodes in experiments described later. However, it is notable that this variability does not change as a function of the age of the SPAAC P–GFF reaction mixture (Fig. 4(a)), with no correlation between *R*_ct_ and the age (days post click reaction) of the SPAAC P–GFF reaction mixture. This dataset is comprised of extracted *R*_ct_ values from different Au-SPEs modified with the same aliquot of SPAAC P–GFF reaction mixture and demonstrates the remarkable stability of the P–GFF conjugate, which yields self-assembled monolayers over an approximate 10 months time scale despite multiple freeze–thaw cycles. In addition, the formation and stability of the P–GFF conjugate was confirmed by comparison with the EIS response for Au-SPE modified with thiol-polymer only (Fig. S6†). In these ‘GFF-free’ control experiments a significantly larger Nyquist response is observed for the ‘polymer-only’ dataset (average *R*_ct_ = 89.5 kΩ). This is attributed to differences in the interactions of the solution-based ferricyanide with polymer (P) *versus* polymer–glycofluoroform (P–GFF); the anionic charge of the Fe(CN)_6_^4−/3−^ redox couple makes it highly sensitive to changes in electrode surface electrostatics.^52^ The difference between the control experiment data and data from the modification of different Au-SPE with a single P–GFF aliquot (Fig. 4(a)) supports our assertion that the glycofluoroform modified polymer is stable over a prolonged timescale.

To further assist in the visualisation of differences between datasets, Fig. 4(b) presents an overlay of the distributions of *R*_ct_ values from repeat experiments analysing bare Au-SPEs, the full P–GFF dataset (both from Fig. 3(d)), the subset of P–GFF experiments carried out across many different days using the same polymer–glycofluoroform solution to modify the electrode (data from Fig. 4(a)), and polymer-only control experiments (Fig. S6†). Complementary *t*-test analysis (see ESI†) supports the conclusion that there is a statistically significant difference between the unmodified Au-SPE “bare” electrodes and the combined P–GFF dataset, but the inter-day variability is insignificant when this sub-dataset (*N* = 20) is compared to the mean of the collated P–GFF dataset from all experiments (*N* = 63). We did not make comparison to the polymer-only dataset because as shown in Fig. 4(b) we have insufficient data to resolve the skewed distribution in this relatively small (*N* = 5) set of control experiments, and polymer-only modifications are not the focus of this study.

### Galectin-3 binding

**Fig. 5:**
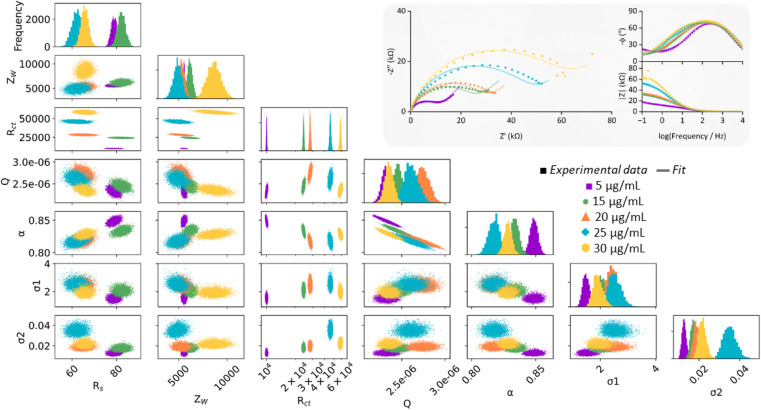
2D scatter plots showing correlations for circuit parameters used to infer parameter distributions from a representative galectin-3 incubation experiment. The diagonal represents the 1D histograms for each parameter showing the distribution of the fitted values. The MCMC used to generate these chains was run for 10 000 samples, with the first 3000 discarded as burn-in. Inset: resultant Nyquist plots (dots) and the computational fitting (line) and Bode plots for the galectin-3 incubation experiments. EIS measurements were performed in the presence of 10 mM K_3_[Fe(CN)_6_] in pH 7 aqueous buffer (100 mM sodium phosphate, 233 mM sodium chloride) with the following parameters: *t*_(equilibration)_ = 180 s, *E*_dc_ = 0.230 V *vs.* Ref, *E*_ac_ = 10 mV, *f* = 0.05 Hz–10 kHz.

Following characterisation of the surface immobilisation of the thiol polymers onto Au-SPEs, we next investigated the ability of the glycofluoroform-decorated SPEs to detect galectin-3 in solution. Galectin-3 titration experiments were performed using EIS analysis, with a 30 minutes incubation time used for each protein concentration based on binding kinetics from previously published work.^32^ A dataset and corresponding MCMC analysis for a single P–GFF modified Au-SPE (Fig. 5) was generated, with fitting performed for each concentration of galectin-3 to find the best fit values for all seven parameters in the equivalent circuit model (five parameters constitute the modified Randles circuit plus two noise parameters for the phase and magnitude of the impedance). Notably, we observed an increase in magnitude of *R*_ct_ with galectin-3 concentration and importantly, as shown in the *R*_ct_ histogram, the distribution of the fitted values of this parameter at different protein concentrations showed no overlap. This data therefore indicated the successful construction of an electrochemical impedimetric biosensor which is responsive to changing levels of galectin-3. In contrast to earlier experiments comparing different electrodes (Fig. 3(b)), there are smaller distributions in the non *R*_ct_ parameters, in particular the *R*_s_ and *Z*_W_ values overlap extensively, suggesting these parameters are insensitive to a change in galectin-3 concentration. Indeed, we recommend such MCMC data visualisation as a powerful tool for identifying a sensible protein concentration range over which to conduct experiments, and validation that *R*_ct_ values from measurements at different analyte concentrations have statistically significant differences.

**Fig. 6:**
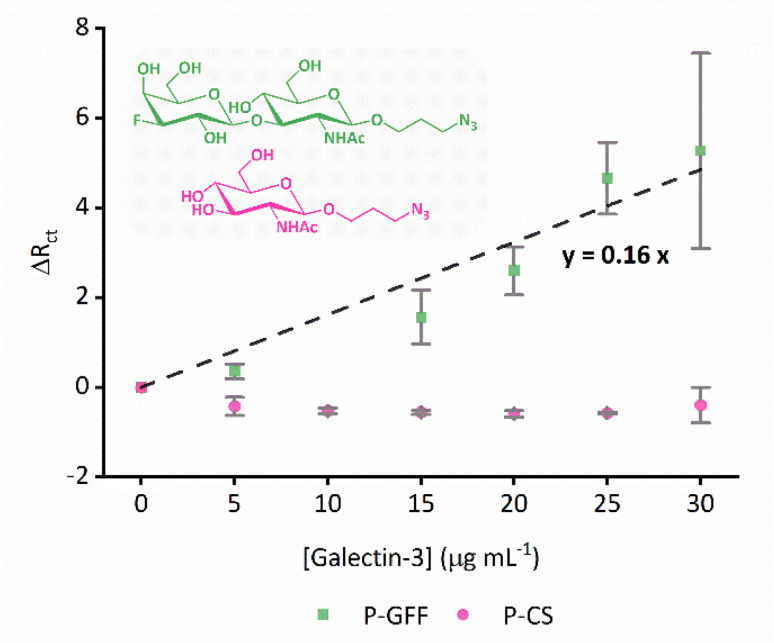
Combined datasets from three different P–GFF (green squares) and P-CS (pink dots) modified Au-SPEs titrated against galectin-3. The P–GFF dataset is fit to a straight line (black dashed line, eqn (1)) to enable determination of *K*_d_, as described in the text. Inset: chemical structures of GFF (green) and CS (pink). The datapoints represent the average values from the combined datasets, while vertical error bars represent the standard deviation.

Given the substantive variation shown between P–GFF modified Au-SPEs, we repeated the galectin-3 titration experiments on a total of 15 different P–GFF modified SPEs (Fig. S7†), noting a general trend of increasing *R*_ct_ with increasing galectin-3 across a protein concentration range of 15–30 μg mL^−1^ (Fig. S8†). MCMC analysis was used to identify three electrodes which had sufficiently similar *R*_ct_ values in the initial protein-free EIS experiments (12.9, 7.7, and 10.4 kΩ) to justify amalgamation (Fig. S8†). The *R*_ct_*vs.* galectin-3 concentration plot for this combined dataset is shown in Fig. S9(a),† and this confirms that a reproducible galectin-3 binding response is achieved across repeat electrodes where they have a similar initial *R*_ct_. Applying the analysis described by Bandyopadhyay *et al.*,^53^ assuming a Langmuir isotherm, enables the dataset to be linearised as shown in Fig. 6 with the gradient of the plot corresponding to 1/*K*_d_ as shown in eqn (1), where *C* is the galectin-3 concentration and *R*_ct,0_ and *R*_ct,i_ are the resistance to charge transfer at a protein-free stage and after protein incubation, respectively. Using this analysis and setting a *y*-intercept of zero yields a *K*_d_ of 6.3 μg mL^−1^ which equates to 240 nM assuming a molecular weight of 26 kDa for galectin-3. The *K*_d_ value for the GFF EIS biosensor is notably higher than the *K*_d_ of 6.0 nM achieved using the same P–GFF binding system on Au nanoparticles,^32^ we attribute this to the differences in the grafting density and the increased steric repulsion in the interaction between solution-phase galectin-3 and a planar Au-SPE surface compared to a solution suspension of Au nanoparticles.1
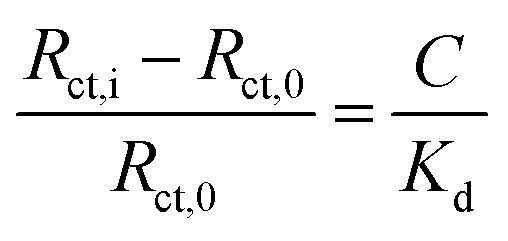


An alternative method of assessing ligand-protein binding affinity is to use the Hill equation, as has been previously reported in the electrochemical biosensor literature.^54–56^ As shown in Fig. S9(b),† such an analysis can also be used to generate a good fit to the data. The best-fit Hill coefficient value, *n* = 5.7, is consistent with the notion that the proposed multimeric–multivalent binding mode of galectin-3 (illustrated in Fig. 1) supports cooperative binding between the protein and the P–GFF surface.^35^ The best-fit value for *K*_A_, the ligand concentration producing half occupation, was 18.5 μg mL^−1^ (equivalent to 713 nM).

### Control experiments

To confirm that the observed galectin-3 concentration dependent response is a result of the specific affinity of the protein for the 3F-lacto-*N*-biose glycofluoroform, further protein titration experiments were carried out using a non-galactoside control monosaccharide with negligible affinity for galectin-3 (GlcNAc-N_3,_Fig. 6). Once again, the DBCO polymer was subjected to SPAAC with the azide containing monosaccharide, before the polymer-control sugar (P-CS) modified electrodes were incubated with galectin-3. A non-concentration dependent *R*_ct_ response to galectin-3 was seen across three P-CS modified Au-SPEs (Fig. S10†) indicating that binding of galectin-3 to modified electrodes is glycofluoroform specific. To best summarise this, the EIS response for P-CS modified electrodes was compared to P–GFF electrodes using the *K*_d_ analysis described above, as shown in Fig. 6.

**Fig. 7:**
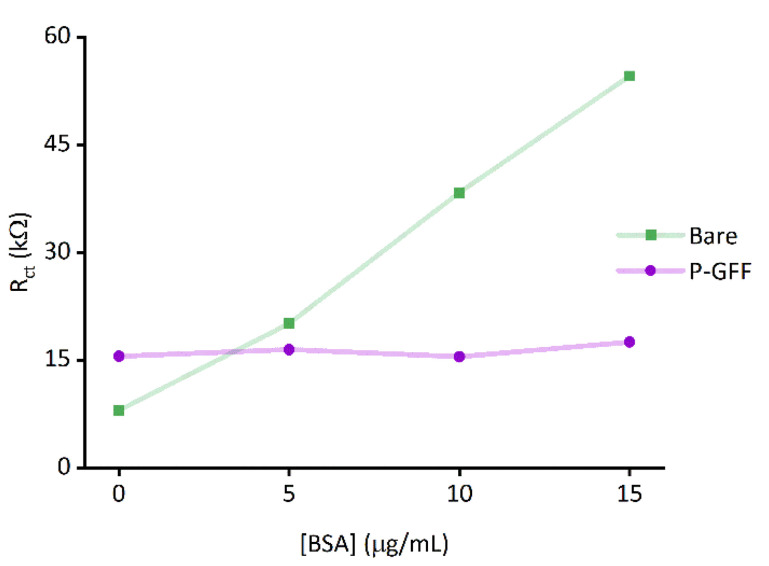
Extracted *R*_ct_ parameters for BSA incubation of both bare and P–GFF modified Au-SPEs.

Finally, an ideal diagnostic biosensor would show minimal off-target binding to other biomolecules present in biological samples; this can be very challenging to achieve because serum contains albumins and immunoglobins, and plasma additionally contains fibrinogen^57^ which are all proteins capable of non-specific surface-binding interactions. We therefore explored whether the incorporation of the P–GFF within the biosensor would act to advantageously reduce any non-specific binding, using albumin (BSA) as a model analyte. Importantly, although EIS experiments using increasing concentrations of BSA (Fig. 7) show a significant change in *R*_ct_ on bare electrodes, this non-specific binding to the gold electrode is reduced when using electrodes modified with the P–GFF.

## Conclusions

This work establishes how the enhanced protein-binding specificity of a glycofluoroform can be harnessed for electrochemical biosensing. The potential of this platform is showcased in the detection of galectin-3 using disaccharide glycofluoroform modified Au-SPEs, establishing charge transfer resistance as a proxy measurement for protein binding. By detecting a biomedically relevant protein, we aim to demonstrate the potential for long-term diagnostic device development. The electrode surface modification ensures sensitivity and selectivity for the target analyte, the glycofluoroform–polymer also exhibits robust stability over a prolonged period, an advantage over other less chemically stable/storable biosensing biomolecules,^52,58^ and the P–GFF also pacifies the gold surface to non-specific albumin binding. Although we demonstrate Au-SPEs exhibit electrode-to-electrode variation which must be accounted for during analysis, they are also compatible with low-volume measurements and this enticing combination of electrochemical cell miniaturization with sensitive and specific binding is highly desirable in point-of-care diagnostic applications.^59,60^ We hope that this paper demonstrates the important need for a molecular understanding of how to design and build selective lectin binders. Our future aim is to expand this work to other biologically relevant glycofluoroforms,^28^ and we anticipate that this work will inspire others to utilise such molecules as a new class of bioreceptors, adding a new and powerful tool to the biosensing toolkit.

## Data availability

Data for this article is available at the University of York repository: https://doi.org/10.15124/7d29d86e-ce9a-46d1-b00f-d76f388b57e2.

## Author contributions

Alice R. Hewson – data curation, formal analysis, investigation (lead), methodology, visualisation, writing – original draft, writing – review & editing; Henry O. Lloyd-Laney – data curation, formal analysis, software (implementation and programming support), writing – review & editing; Tessa Keenan – resources, writing – review & editing; Sarah-Jane Richards – resources, writing – review & editing; Matthew I. Gibson – funding acquisition, resources, writing – review & editing; Bruno Linclau – funding acquisition, writing – review & editing; Nathalie Signoret – funding acquisition, project administration, supervision, writing – review & editing; Martin A. Fascione – conceptualisation, funding acquisition, project administration, supervision, writing – original draft, writing – review & editing; Alison Parkin – conceptualisation, formal analysis, funding acquisition, project administration, supervision, writing – original draft, writing – review & editing.

## Conflicts of interest

The authors declare no competing financial interest.

## Supplementary Material

SC-OLF-D4SC04409F-s001
