# Supplementary material for: Harnessing glycofluoroforms for impedimetric biosensing
Source: Chem Sci. 2024 Sep 13;15(39):16086–95. doi: 10.1039/d4sc04409f (PMC11393611; doi:10.1039/d4sc04409f)
Supplement: SC-015-D4SC04409F-s001 [file SC-015-D4SC04409F-s001.pdf]

Supplementary Information (SI) for Chemical Science.
This journal is © The Royal Society of Chemistry 2024

Supporting Information for: Harnessing Glycofluoroforms for Impedimetric Biosensing

Alice R. Hewson,[a] Henry O. Lloyd-Laney,[a] Tessa Keenan,[a] Sarah-Jane Richards,[b, c] Matthew I. Gibson,[b, c] Bruno Linclau,[d, e] Nathalie Signoret,[f] Martin A. Fascione,[a] and Alison Parkin.[a]

[a] Department of Chemistry, University of York, York, UK, YO10 5DD.
[b] Department of Chemistry, The University of Manchester, UK, M13 9PL.
[c] Manchester Institute of Biotechnology, The University of Manchester, UK, M1 7DN.
[d] Department of Organic and Macromolecular Chemistry, Ghent University, Krijgslaan 281-S4, Belgium, 9000 Gent.
[e] School of Chemistry, University of Southampton, Highfield, Southampton, UK, SO17 1BJ.
[f] Hull York Medical School, University of York, York, UK, YO10 5DD.
*alison.parkin@york.ac.uk, *martin.fascione@york.ac.uk, *nathalie.signoret@york.ac.uk

Contents

Supplementary Materials and Methods 2
- Materials and General Procedures 2
- Synthesis of azidopropane 3-fluoro-3-deoxy-β-D-galactopyranosyl-(1❍3)-2-acetamido-2-deoxy- β-D-glucopyronoside (azido 3F lacto-*N*-biose, 3FGal-β(1,3)-GlcNAc-N_3) 3
- Ferrocene Derivative (FD-01) Synthesis 4
- Ferrocene Derivative (FD-01) Au-SPE modification 4
- MCMC Methods 4

Electrochemical comparison of bare Au-SPEs and P-GFF modified Au-SPEs in the absence of protein binding 6
- Electrochemical 'Stripping' 6
- Ferrocene Immobilisation 7
- Bare and P-GFF Full Datasets 8
- Polymer-Only Control Experiments 10
- Statistical Analysis of Repeat Electrode Modifications 10

Galectin-3 Titration Repeats 12
- Full Nyquist Dataset and Fits 12
- Extracted R_{ct} Values 13

NMR Spectra 16

References 19

Supplementary Materials and Methods

Materials and General Procedures

^{1}H-NMR (400 MHz) and ^{19}F-NMR (400 MHz) experiments were conducted using a JEOL 400 instrument at The University York Centre for Magnetic Resonance. Where NMR is reported, multiplicities are given as singlet (s), doublet (d), triplet (t), quartet (q), or multiplet (m). All NMR chemical shifts (δ) were recorded in ppm and coupling constants (J) are reported in Hz. Data processed using MestReNova software.

For structure confirmation, High Resolution Electrospray Ionisation Mass Spectrometry (HRMS) data was obtained at room temperature on a Bruker Daltonics microTOF mass spectrometer coupled to an Agilent 1200 series LC system at The University of York Centre of Excellence in Mass Spectrometry (CoEMS). Nominal and exact m/z values are reported in Daltons.

For reaction monitoring and fraction screening, High Performance Liquid Chromatography-Electrospray Ionisation Mass Spectrometry (LC-ESI-MS) was carried out on a Dionex UltiMate® 3000 LC system (ThermoScientific) in line with a Bruker HCTultra ETD II system (Bruker Daltonics). Analyses were carried out using a SeQuant ZIC®-HILIC (3.5 µM, 200 Å) 100 X 2.1 mm HPLC column. Water, 0.1% formic acid by volume (solvent A), and acetonitrile, 0.1 % formic acid (solvent B) were used as the mobile phase at a flow rate of 0.5 ml/min at room temperature. A multi-step gradient of 9 min was programmed as follows: 95 % B for 0.5 min, followed by a linear gradient to 5 % B over 4.5 min, followed by 5 % B for an additional 0.5 min. A linear gradient to 95 % B was used to re-equilibrate the column.

Analytical thin layer chromatography (TLC) was carried out on pre-coated 0.25 mm Merck KgaA 60 F254 silica gel plates. Visualisation was by using p-Anisaldehyde stain.

Materials were procured from commercial sources and used without further purification unless otherwise stated. Triethylamine, deuterium oxide, magnesium chloride, sodium dihydrogen phosphate, 4-(2-Hydroxyethyl)piperazine-1-ethanesulfonic acid sodium salt (HEPES), Trizma® hydrochloride, and Adenosine-5'-triphosphate (ATP) disodium salt hydrate were purchased from Sigma-Aldrich. Dichloromethane, methanol, n-butanol, ethyl acetate, and sodium chloride were purchased from Fischer Scientific. 11-azido-3,6,9-trioxaundecan-1-amine was purchased from Tokyo Chemical Industries (TCI). Potassium ferricyanide was purchased from Acros Organics. 3-Deoxy-3-fluoro-galactose (3FGal) was provided by Carbosynth. 3-ferrocenyl propanoic acid N-hydroxyphthalimide ester was provided by Dr Nicholas D. J. Yates and synthesised as previously reported. [1] Milli-Q H_2O was obtained from a Purite HP 320 water purification system.

Synthesis of azidopropane 3-fluoro-3-deoxy-β-D-galactopyranosyl-(1O3)-2-acetamido-2-deoxy- β-D-glucopyronoside (azido 3F lacto-*N*-biose, 3FGal-β(1,3)-GlcNAc-N_3)

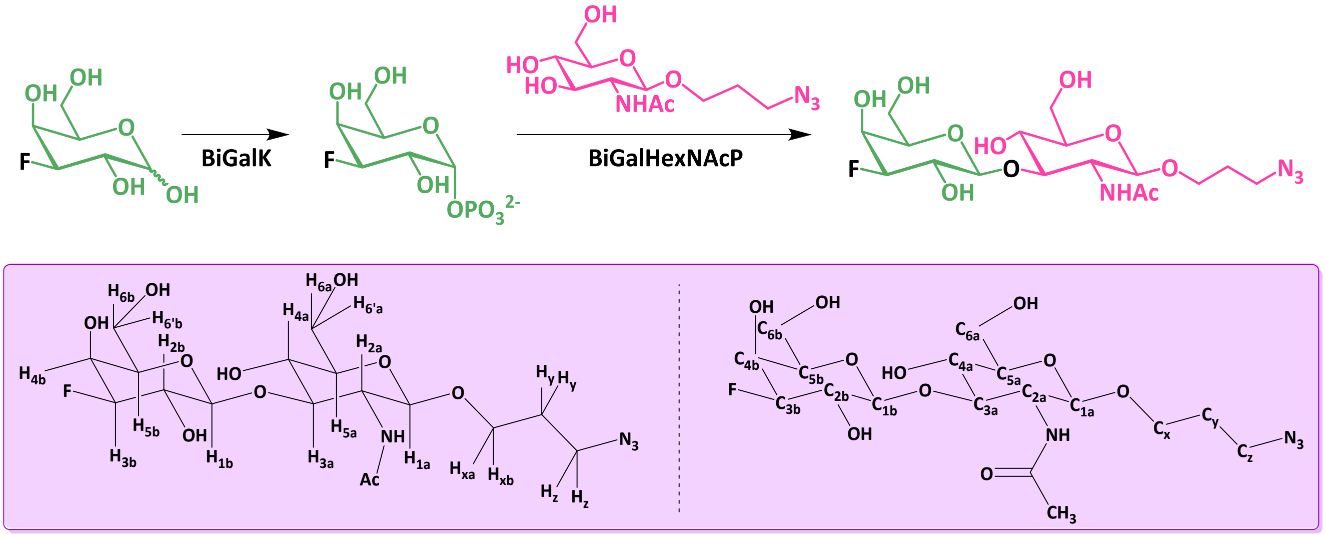

Scheme S1. *Reaction scheme for the BiGalK-catalysed phosphorylation of 3F-galactose followed by the BiGalHexNAcP-catalysed transfer of phosphorylated galactose to a N-acetylglucosamine acceptor by reverse phosphorylysis yielding the fluorinated disaccharide, 3FGal-β(1,3)-GlcNAc-N_3 (GFF).*

3FGal-β(1,3)-GlcNAc-N_3 was synthesised as previously reported,[2] but in a modified two-step approach as this was found to be higher yielding. A reaction containing 3FGal (8 mM, 91 mg), ATP (10 mM, 344 mg), $MgCl_2$ (5 mM, 3125 µL of a 100 mM stock), Tris HCl buffer pH 8.0 (100 mM) and BiGalK (0.12 mg mL^{-1}, 7.496 mg) was assembled in a sterile pot to a final reaction volume of 62.5 mL, and adjusted to pH 8.0. Following mixing, the reaction was separated into 2 mL aliquots and incubated at 37 °C for 24 h. Completion of the reaction was validated by TLC (R_f = 0.34, N-butanol, acetic acid, H_2O 2:1:1). Aliquots were subsequently pooled and BiGalK was removed using a centrifugal spin concentrator (10 kDa MWCO) and lyophilised. For the second step the reaction was assembled on a 2000 µL scale in a sterile pot containing 100 mM Tris. HCl buffer (pH 6.5), crude 3FGal1P (25 mM, 13 mg), acceptor monosaccharide, GlcNAc-N_3, (10 mM, 6.1 mg), $MgCl_2$ (15 mM, 2.9 mg) and BiGalHexNAcP (42.3 mg). Following mixing, the reaction was separated into 500 µL aliquots in 2 mL Eppendorfs and incubated at 37 °C. Reaction progress was monitored via TLC (R_f = 0.66 N-butanol, acetic acid, H_2O 2:1:1) and LC-MS and additional BiGalHexNAcP (2.12 mg) was added to each aliquot every 24 hours until completion. Reaction mixtures were pooled and BiGalHexNAcP was removed using a centrifugal spin concentrator (10 kDa MWCO) and the sample was lyophilised to yield the crude disaccharide. The crude disaccharide was then purified in a two-step process. First, using a CombiFlash® NextGen system (Teledyne) purification system, eluting with a EtOAc-MeOH gradient, and then size-exclusion chromatography (Biogel P2) eluting with dH_2O. The purified disaccharide was characterised by ^{1}H, ^{13}C, and ^{19}F NMR and MS.

^{1}H NMR (400 MHz, D_2O) δ 4.60 (ddd, *J* = 10.0, 3.7, 1.3 Hz, 1H, H-3b), 4.53 (d, *J* = 7.7 Hz, 1H, H-1b), 4.46 (d, *J* = 7.8 Hz, 1H, H-1a), 4.21 – 4.16 (m, 1H, H-4b), 3.97 (ddd, *J* = 10.6, 5.7, 1.7 Hz, 1H, H-xa), 3.91 (dt, *J* = 12.6, 2.2 Hz, 1H, H-6a), 3.87 – 3.60 (m, 8H, H-2a, H-2b, H-4a, H-5b, H-6a’, H-6b, H-6b’, H-xb), 3.57 – 3.50 (m, 1H, H-3a), 3.50 – 3.40 (m, 1H, H-5a), 3.35 (td, *J* = 6.5, 3.2 Hz, 2H, H-z), 2.04 – 2.00 (m, 3H, NHAc), 1.89 – 1.78 (m, 2H, H-y).

^{13}C {^{1}H) NMR (100 MHz, D_2O) δ 174.61 (C=O), 102.86 (C-1a), 100.93 (C-1b), 93.65 (C-3b), 82.48, 73.95, 69.46, 69.27, 60.72 & 54.61 (C-2a, C-2b, C-4a, C-5b, C-6a, C-6b), 75.39 (C-5a), 68.68 (C-3a), 67.19 (C-x), 66.69 (C-4b), 47.81 (C-x), 28.12 (C-y), 22.21 ($NHCOCH_3$).

^{19}F {^{1}H} NMR (400 MHz, D_2O) δ -198.95.

HRMS (ESI): $C_{17}H_{29}FN_4NaO_{10}$ $[M+Na]^+$ Calculated: 491.1760; Found: 491.1755 (mean error 0.6 ppm). $C_{17}H_{28}FN_4O_{10}$ $[M-H]^-$ Calculated: 467.1795; Found: 467.1816 (mean error 0.9 ppm).

Ferrocene Derivative (FD-01) Synthesis

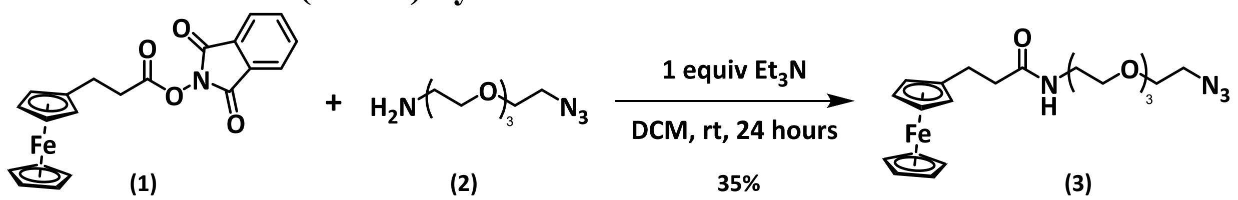

Scheme S2. *Synthesis of FD-01 (3).*

3-Ferrocenyl propanoic acid N-hydroxyphthalimide ester (1) (5 mg, 12.4 µmol, 1.0 equiv), 11-azido-3,6,9-trioxaundecan-1-amine (2) (13.6 6 µmol, 1.1 equiv), and triethylamine (1.73 µL, 1.0 equiv) were dissolved in DCM (100 µL) and stirred at room temperature for 24 hours. The solvent was removed in vacuo. The resultant solid was semi-purified via silica flash column chromatography (starting with DCM + 1% Et_3N and moving to DCM + 1% Et_3N + 2% MeOH once the first band had eluted) to yield an orange solid of the ferrocene derivative, FD-01, (3); 3.7 mg, 8.06 µmol, 65%.

HRMS (ESI): $C_{21}H_{31}FeN_4O_4$ $[M+H]^+$ Calculated: 459.1689; Found: 459.1695 (mean error -2.2 ppm).
$C_{21}H_{30}FeN_4NaO_4$ $[M+Na]^+$ Calculated: 481.1509; Found: 481.1520 (mean error -1.3 ppm).

Ferrocene Derivative (FD-01) Au-SPE modification

FD-01 and the thiol polymer undergo an analogous SPAAC reaction to that described between thiol polymer and sugar in materials and methods. Following the SPAAC reaction, 450 µL aliquots of the reaction mixture in 2 mL Eppendorf tubes were used to modify Au-SPEs simultaneously by immersing the SPEs to a depth where all electrodes were covered and leaving them at room temperature in an anaerobic glovebox environment for 18 – 24 hours. The modified electrodes were then analysed by DCV, experimental conditions are provided in the figure captions.

MCMC Methods

The Gaussian log-likelihood function is defined as follows for a set of equivalent circuit parameters *p* that are combined to predict an impedance response *f(p)* at *n* frequencies *ω,* and compared to impedance data ***x*** (both the model and data are in their polar forms, and so take the form of a 2xn matrix). We assume an independent joint likelihood for the phase and magnitude, and assume the noise in both variables is identically and independently distributed. This results in the following log-likelihood

$$L\,(p, \sigma\,|x) = -\,n\,log2\pi - \sum_{i=1}^{2} n log(\sigma_i) - \sum_{i=1}^{2}\left[\frac{1}{2\sigma_i^{\,2}}\sum_{j=1}^{n}(x_{i_j} - f_{i_j}(p))^2\right]$$

Table S1. *RHat value for the MCMC chains plotted in figure 3 and figure 5 in the main paper, as implemented in the PINTS repository. This is a heuristic to assess the level of convergence between independent chains (after burn-in has been discarded) for each parameter. For values under 1.1, the chains are judged to have converged.*

Fig #	Repeat #	[Galectin-3] / µg mL^{-1}	RHat value for the MCMC chains						
			R_s	Z_W	R_{ct}	Q	α	σ1	σ2
3	1	0	1.005	1.009	1.011	1.009	1.008	1.016	1.003
	2	0	1.007	1.003	1.004	1.005	1.004	1.001	1.009
	3	0	1.004	1.008	1.008	1.005	1.006	1.003	1.005
	4	0	1.001	1.005	1.002	1.001	1.001	1.004	1.007
	5	0	1.002	1.004	1.004	1.002	1.002	1.006	1.001
	6	0	1.002	1.002	1.006	1.007	1.007	1.001	1.008
	7	0	1.001	1.003	1.003	1.003	1.003	1.004	1.001
	8	0	1.002	1.006	1.002	1.002	1.002	1.004	1.003
5	12	5	1.003	1.001	1.001	1.001	1.000	1.005	1.008
	12	15	1.002	1.002	1.003	1.004	1.004	1.009	1.002
	12	20	1.001	1.003	1.004	1.001	1.001	1.010	1.011
	12	25	1.002	1.002	1.001	1.002	1.003	1.006	1.006
	12	30	1.001	1.004	1.003	1.002	1.003	1.005	1.002

Table S2. *Parameter bounds used for both the CMA-ES and MCMC algorithms (in the form of a log prior for the latter).*

Parameter	Lower Bound	Upper Bound
R_s / Ω	0	1×10^4
W / Ω $s^{-½}$	0	1×10^6
R_{ct} / Ω	1×10^{-6}	1×10^6
Q	0	2
α	0	1

Electrochemical comparison of bare Au-SPEs and P-GFF modified Au-SPEs in the absence of protein binding

Electrochemical 'Stripping'

Electrochemical evidence for the presence of gold-thiol bond formation following incubation in SPAAC P-GFF reaction mixture or polymer-only (P) solution can be seen in 'stripping experiments' (**Figure S1(a) and (b)**). By carrying out direct current cyclic voltammetry (DCV) over a wide potential range, we activate cleavage of the gold-thiol bonds and the CV signal returns to that of a bare Au-SPE. This is substantiated by work in the literature which shows that applying a negative potential to a gold surface causes reduction of the thiol bonds, a process known as electrochemical desorption.[3] In addition, comparison of the Bode plot before and after a 'stripping' experiment (**Figure S1(c)**) indicates the removal of immobilised substrate due to a large decrease in phase angle.

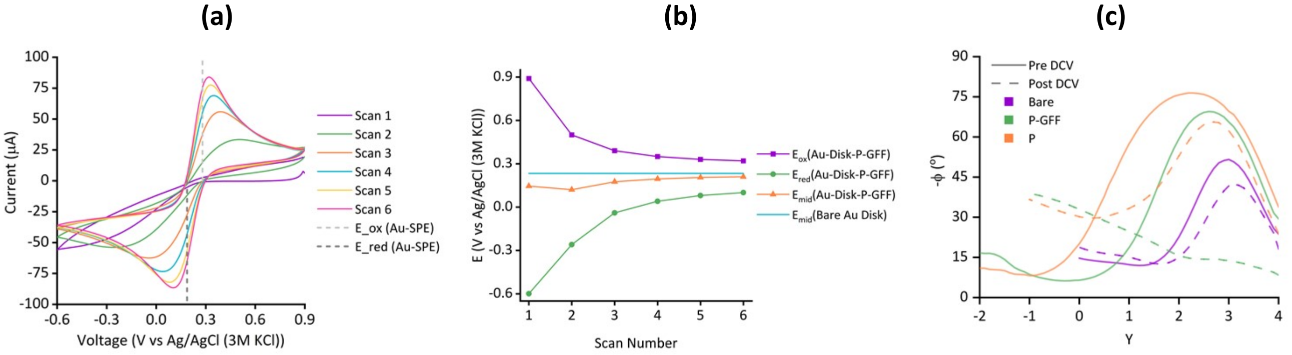

***Figure S1. (a)** 100 mV s^{-1} DCV 'stripping' of P-GFF modified Au disk electrodes; changes in the voltammetric shape arise from electrochemically activated gold-thiol bond cleavage. **(b)** Analysis of the peak position of data from **(a)**. **(c)** Simplified Bode plots for bare, P modified, and P-GFF modified Au-SPEs pre and post DCV 'stripping'. For EIS experiments, the applied voltage was determined from the midpoint potential of the initial DCV experiment, with an amplitude of 10 mV and an equilibration time of 300 s. The highest frequency for all experiments was 10 kHz, the lowest frequency was either 1, 0.1 or 0.01 Hz. Both DCV and EIS measurements were performed in the presence of 10 mM $K_3[Fe(CN)_6]$ in pH 7 aqueous buffer (100 mM sodium phosphate, 233 mM sodium chloride).*

Ferrocene Immobilisation

The DCV response for surface immobilised ferrocene derivative FD-01 shows a reversible redox process (**Figure S2, green line**). Through baseline fitting and subtraction, the Faradaic current contribution can be isolated (**Figure S2**) and the surface coverage determined to be 0.350 pmol (**Table S3**).[4]

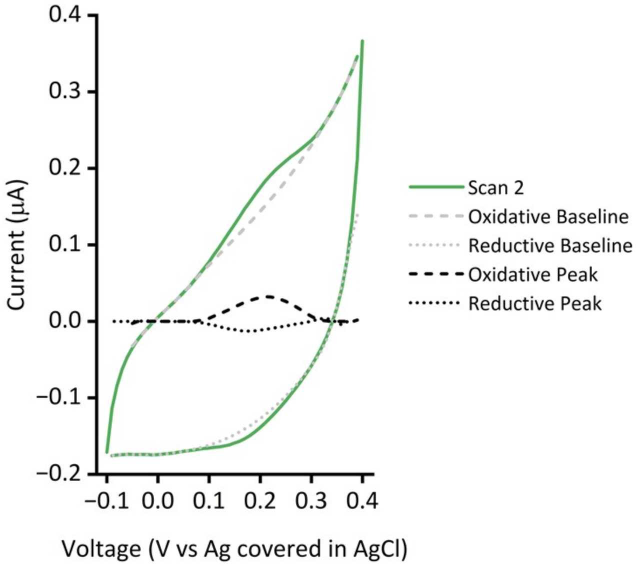

Figure S2. *DCV for immobilised P-FD-01 on an Au-SPE, scan 2 with fitted polynomial baselines and extracted Faradaic peak for both the oxidative and reductive scans is shown. The voltage was cycled between -0.10 V to 0.40 V (vs Ag covered in AgCl) for 5 scans at a scan rate of 100 mV s^{-1}.*

Table S3. *Surface coverage extracted for immobilised FD-01 for oxidative and reductive scans.*

	Oxidative Scan	Reductive Scan
Extracted peak area / A V	4.42×10^{-9}	2.34×10^{-9}
Charge passed / C	4.42×10^{-8}	2.34×10^{-8}
Immobilised FD-01 / pmol	0.458	0.243
Surface coverage / pmol cm^{-2}	14.6	7.72
Average immobilised FD-01 / pmol	0.350	
Average surface coverage / pmol cm^{-2}	11.2	

The maximum polymer coverage, and therefore sugar presentation, is estimated from determining the molecular footprint of the polymer based on Chimera modelling (**Figure S3**). If the polymer 'footprint' is considered to be circular the diameter of the polymer can be calculated as 24.38 Å by approximating the extreme points as an isosceles triangle. The geometric surface area of the working electrode is approximately 0.03 cm^2 so assuming hexagonal close packing (90.7 %), a monolayer polymer coverage can be estimated as 1.01 pmol, or 32.3 pmol cm^{-2}.

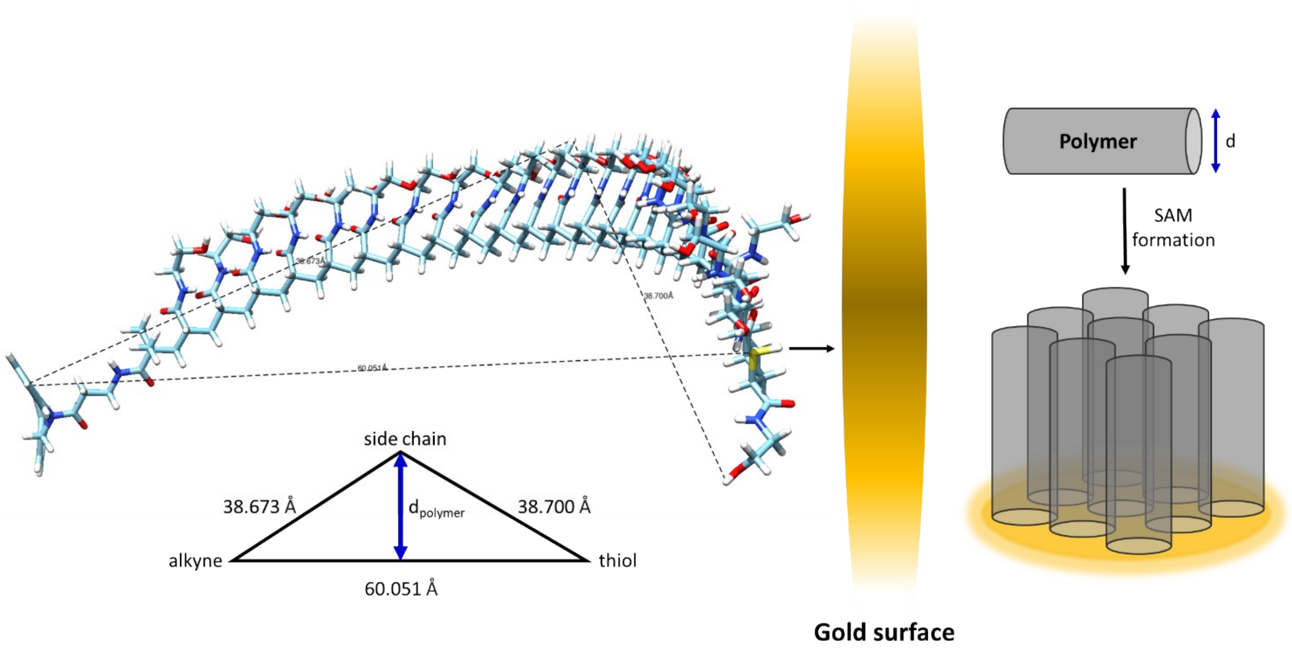

Figure S3. *Predicted 3-D polymer structure and calculated distances (from Chimera) and a schematic showing how an estimated diameter and packing efficiency can be used to calculate a maximum surface coverage estimate.*

Bare and P-GFF Full Datasets

Many different bare and P-GFF modified Au-SPE were interrogated using EIS and the resulting data was analysed by fitting to a modified Randles circuit as shown in **Figure S4** and **Figure S5**. The extracted R_{ct} values from the data fitting were used to generate the box and whisker plot in **Figure 3(d)** in the main manuscript.

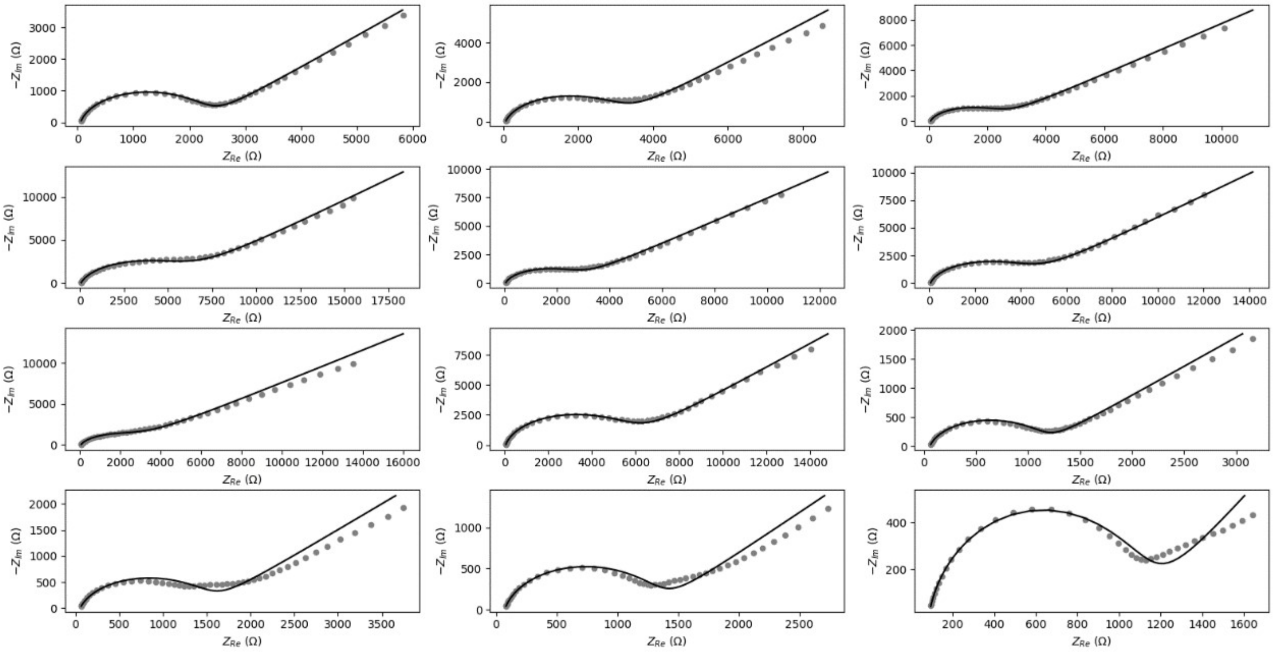

Figure S4. *Nyquist plots (experimental datapoints in grey and fits shown as black lines) for all the bare Au-SPE datasets included in Figure 3(d).*

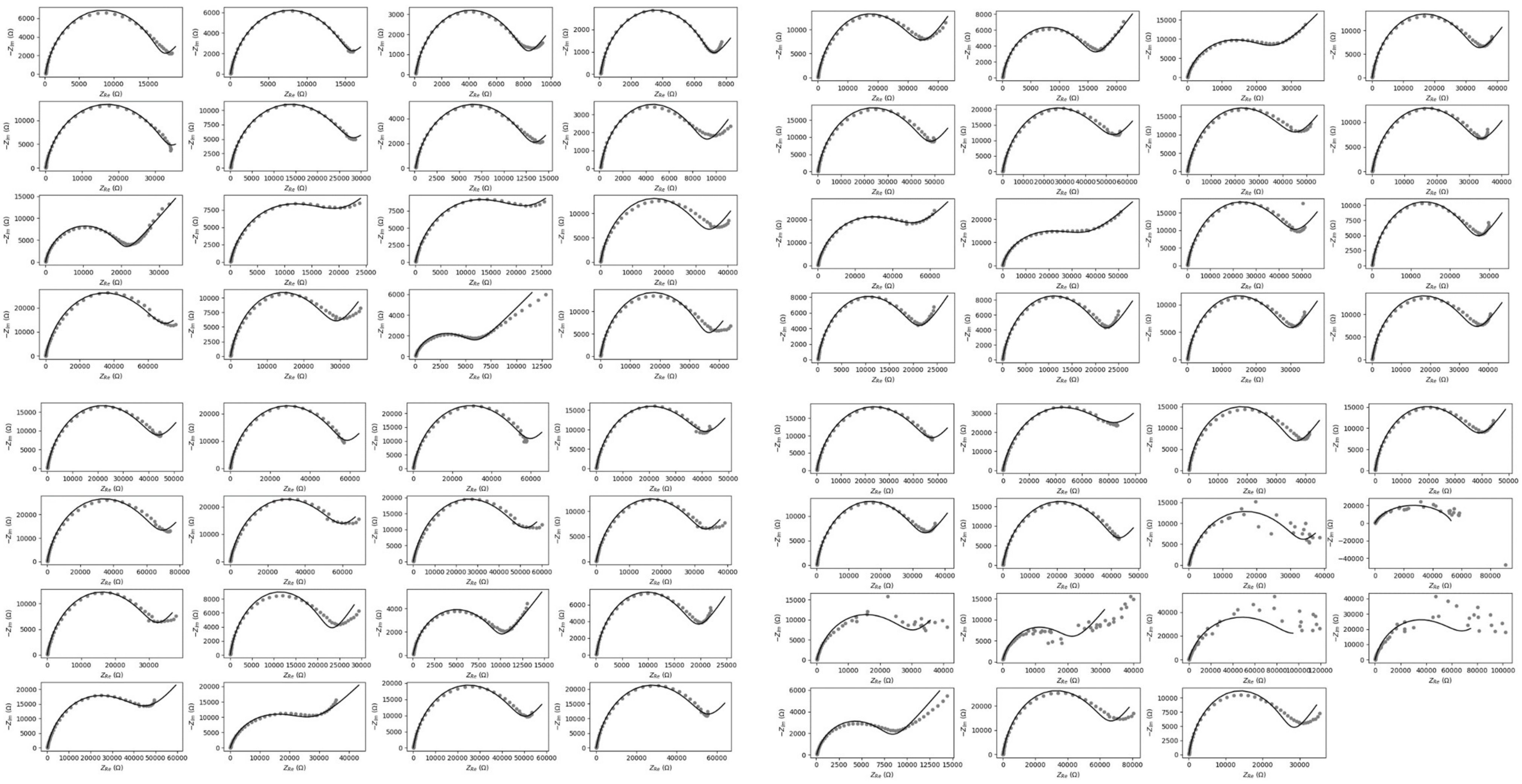

Figure S5. *Nyquist plots (experimental datapoints in grey and fits shown as black lines) for all the P-GFF datasets included in Figure 3(d).*

Polymer-Only Control Experiments

A series of experiments were conducted on ‘polymer only’ modified Au-SPEs where a larger Nyquist response (**Figure S6**) is seen compared to that of P-GFF modified Au-SPEs (**Figure 3(a) and (d)**).

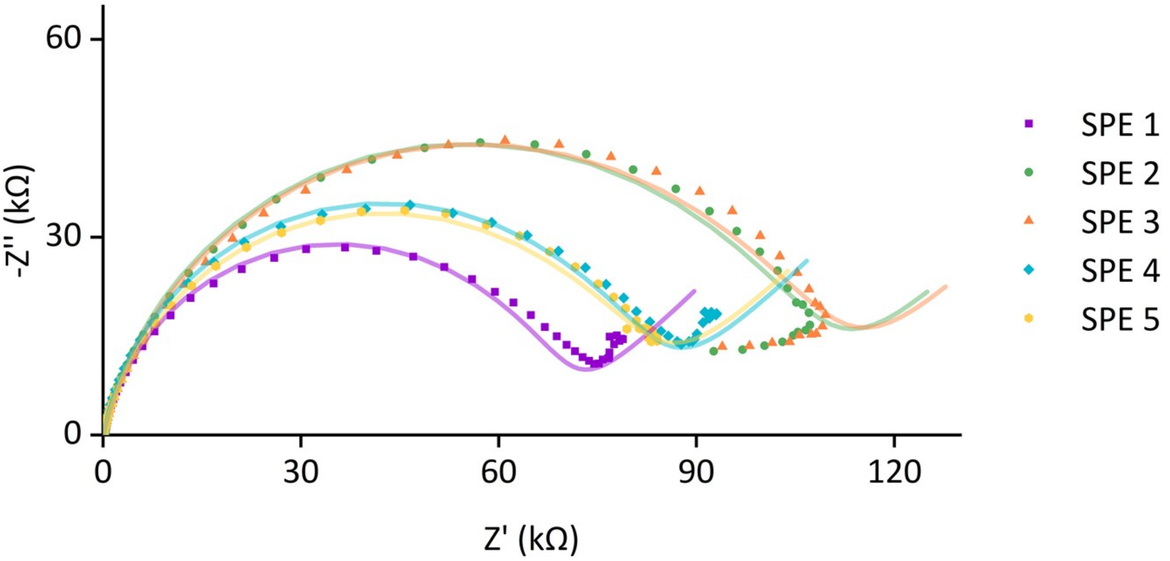

Figure S6. *The electrochemical response of different Au-SPEs modified with just thiol-terminated dibenzocyclooctyne-functionalised poly(hydroxyethyl acrylamide) (DBCO-(PHEA)$_{25}$-SH); i.e. “polymer-only”, GFF-free controls, where the dots are the experimental data and the lines are the computational fits to a modified Randles circuit, the extracted R_{ct} values for the five repeats are as follows: 68.8, 107, 109, 81.8, and 81.0 kΩ.*

Statistical Analysis of Repeat Electrode Modifications

The statistics hypothesis testing tool in Origin (v. 2019b) was used to prove that we can be confident that the bare electrode dataset is significantly different from the data for P-GFF modified Au-SPEs. Conversely, the sub-dataset of measurements on P-GFF electrodes modified with the same solution over a long period of time is not significantly different from the mean of R_{ct} measurements taken from all P-GFF experiments.

- **Two Sample t Test Comparing Bare (N = 16) to P-GFF (N = 63)**

Descriptive Statistics

		N	Mean	SD	SEM	Median
"R\-(ct)"		63	34.1882	17.93713	2.25987	31.78281
		16	2.80768	1.51653	0.37913	2.72767
	Difference		31.38052		4.50985	
	Overall	79	27.83266	20.42722	2.29824	26.93427

Standard Error of Mean (SEM) of difference is computed under the condition that equal variance is assumec

t-Test Statistics

	t Statistic	DF	Prob>|t|
Equal Variance Assumed	6.95822	77	9.9725E-10
Equal Variance NOT Assumed (Welch Correction)	13.69462	65.32533	7.17624E-21

Null Hypothesis: mean1-mean2 = 0
Alternative Hypothesis: mean1-mean2 <> 0
At 0.05 level, when equal variance is assumed, Mean1 - Mean2 is significantly different from 0
At 0.05 level, when equal variance is NOT assumed, Mean1 - Mean2 is significantly different from C

Confidence Intervals for Mean

Conf. Levels in %	Lower Limits	Upper Limits
90	23.87215	38.8889
95	22.40027	40.36078
99	19.46912	43.29192

Plots

Histogram	Box Plot

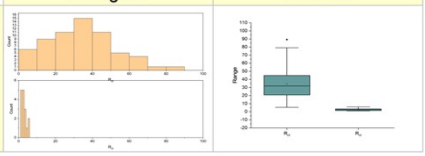

- **One Sample t Test Comparing Inter-Day Variability (N = 20) to the P-GFF Population Mean**

One Sample t Test (27/08/2024 16:44:07

Descriptive Statistics

	N	Mean	SD	SEM
"R\-(ct)"	20	32.15328	13.92686	3.11414

Test Statistics

	t Statistic	DF	Prob>|t|
"R\-(ct)"	-0.65345	19	0.5213

Null Hypothesis: Mean = 34.1882
Alternative Hypothesis: Mean <> 34.1882
"R_{ct}": At the 0.05 level, the population mean is NOT significantly different from the test mean (34.1882).

Confidence Intervals for Mean

	Conf. Levels in %	Lower Limits	Upper Limits
"R\-(ct)"	90	26.76852	37.53804
	95	25.63531	38.67125
	99	23.24393	41.06263

Plots

Histogram	Box Plot
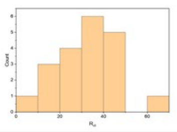	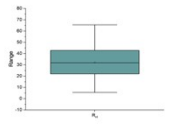

Galectin-3 Titration Repeats

Full Nyquist Dataset and Fits

In order to work out the correct protein concentration range over which P-GFF modified Au-SPEs could be used to sense galectin-3, and to also quantify electrode-to-electrode variation, the fifteen different sets of experiments shown in **Figure S7** were conducted, and all analysed by equivalent circuit fitting.

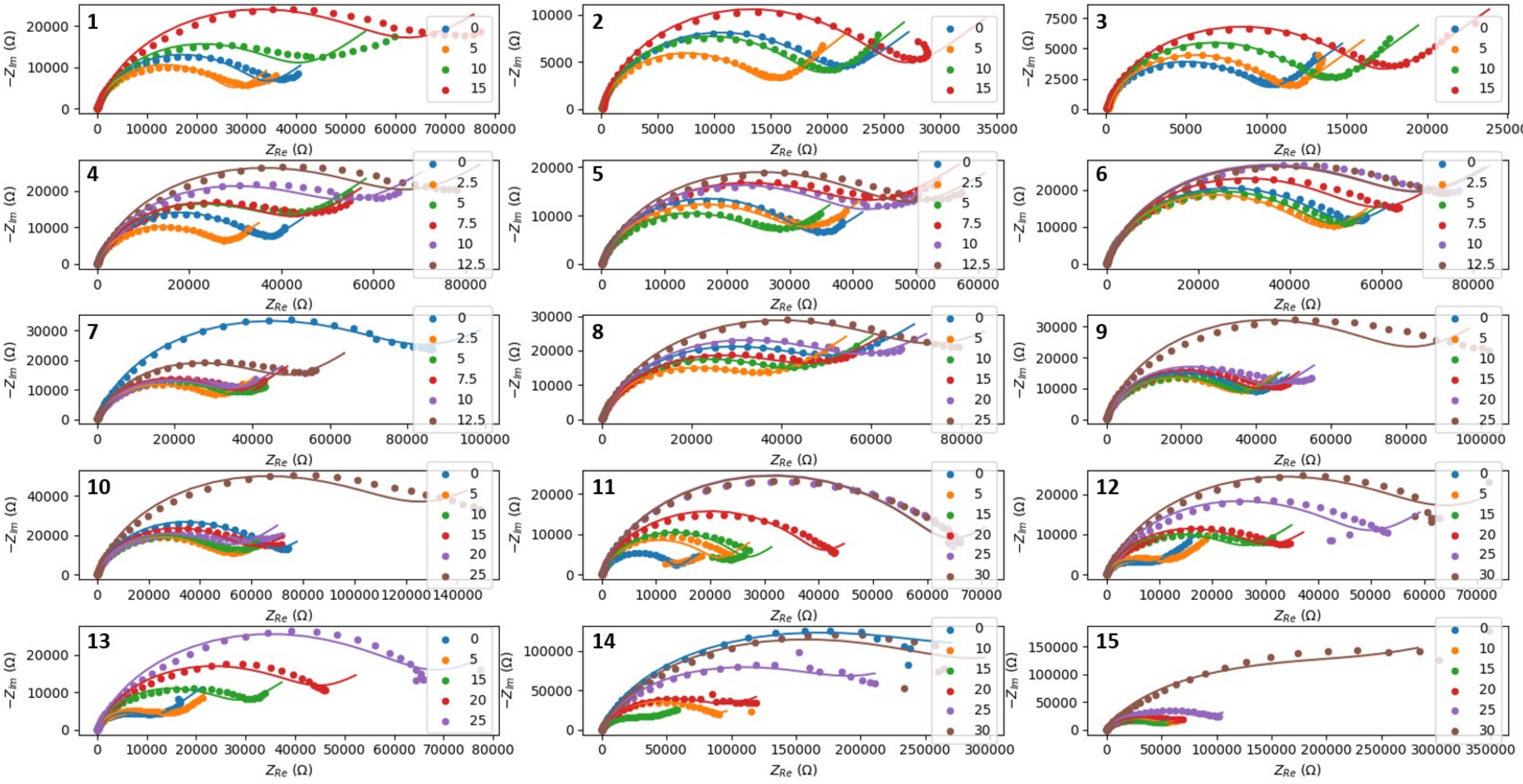

Figure S7. *Nyquist plots, where the dots are the experimentally measured datapoints and the lines are the equivalent circuit fits, for galectin-3 titration experiments performed on different P-GFF modified Au-SPEs, the number is the top-left corner corresponds to the repeat number referenced in* ***Figure S8****.*

Extracted R_{ct} Values

Figure S8(a) shows the extracted R_{ct} values (plotted on a logarithmic scale) for the P-GFF modified Au-SPE galectin-3 titration data from **Figure S7**. In **Figure S8(a)**, data collected over too low a galectin-3 concentration range to permit further analysis, repeats 1 – 7, and datasets with an anomalous initial protein-free measurement (outside the 5th – 95th percentile range from **Figure 3(d)**), are shown in grey. **Figure S8(b)** shows the distribution in R_{ct} values obtained from MCMC analysis of the experiments depicted in black in **Figure S8(a)**.

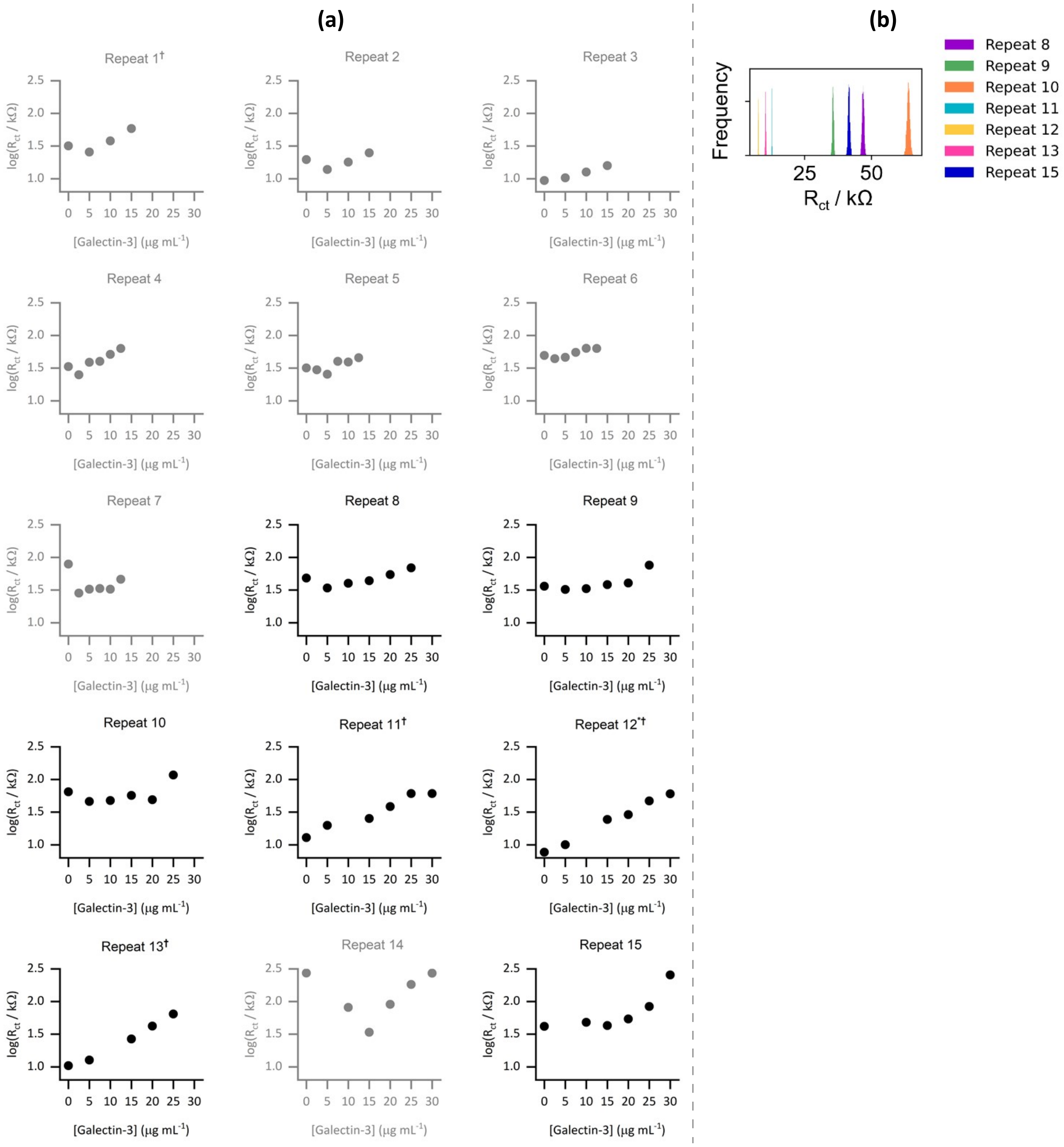

Figure S8. *Extracted R_{ct} parameters for the experiments shown in* ***Figure S7****. (a) Best-fit point values obtained from CMA-ES analysis. * denotes the dataset shown in* ***Figure 5*** *and repeats marked with a † are the datasets combined in* ***Figure 6****. (b) Distributions obtained from MCMC analysis.*

Figure S9(a) is an overlay plot of the three P-GFF galectin-3 titration experiments with the most similar initial 'protein-free' measurements, as judged from the MCMC analysis shown in **Figure S8(b)** (datasets are denoted † in **Figure S8(a))**. The same datasets are also analysed to generate **Figure 6** in the main paper which shows a Langmuir analysis. An alternative analysis of ligand binding is shown in **Figure S9(b)** where the combined dataset from (a) is fit to the Hill equation (shown below) to generate values for the Hill coefficient, n = 5.7, which is usually interpreted as a measure of binding cooperativity, and the ligand concentration producing half occupation, K_A = 18.5 µg mL^{-1} (equivalent to 713 nM).[5–7]

$$\frac{\Delta R_{ct}}{\Delta R_{ct,\,max}} = \frac{[Gal-3]^n}{{K_A}^n + [Gal-3]^n} \quad \text{Hill Equation}$$

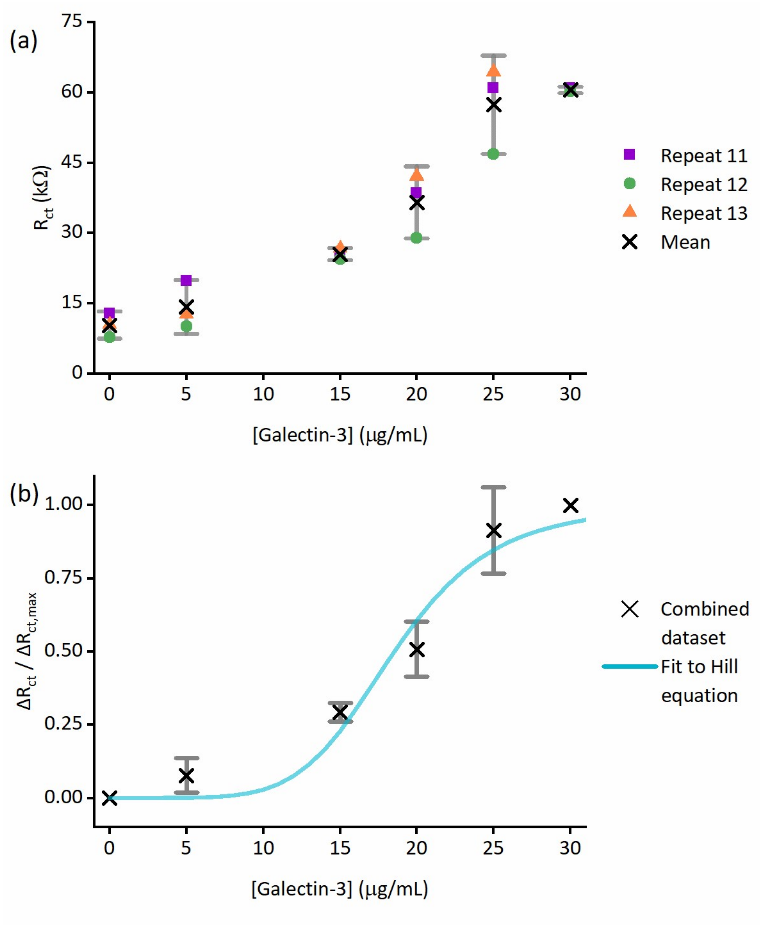

Figure S9. *(a) Extracted R_{ct} parameter plotted against galectin-3 concentration for three SPEs with similar values of protein free initial measurements, the mean value and 95% confidence limits are shown with a black cross and grey vertical error bars, respectively.* ***.*** *(b) Analysis of the combined response-versus-protein concentration dataset (a), where datapoints (black crosses) are the average value from the dataset and vertical error bars denote the standard deviation. A best fit to the Hill equation is shown by the blue line, as described in the text. Here, n =5.7 and K_A (sometimes referred to as the "EC_{50}" value) = 18.5 µg mL-1 (equivalent to 713 nM).*

Figure S10 shows the extracted R_{ct} values (plotted on a logarithmic scale) for the P-CS modified Au-SPE galectin-3 titration data. These datasets are also analysed to generate **Figure 6** in the main paper.

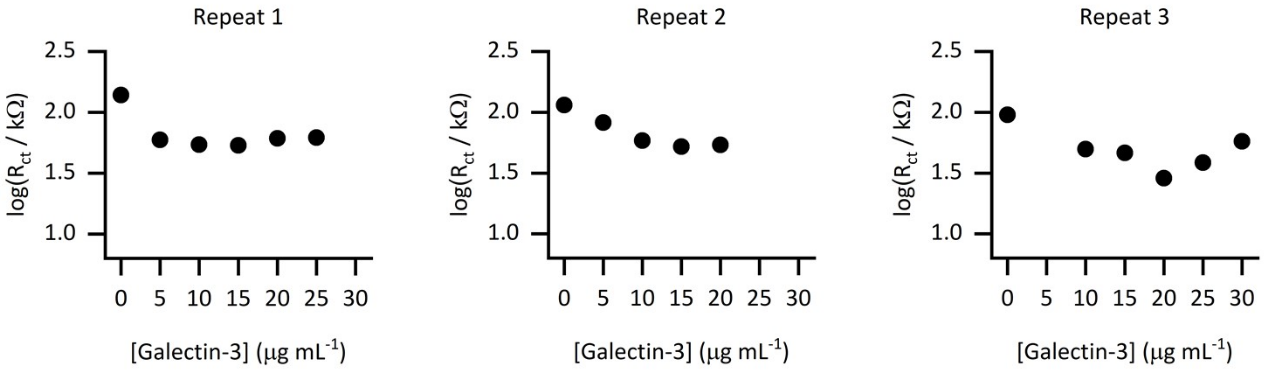

Figure S10. *Extracted R_{ct} parameters for the P-CS modified Au-SPEs.*

NMR Spectra

^{1}H NMR Spectrum for 3FGal-β(1,3)-GlcNAc-N_3

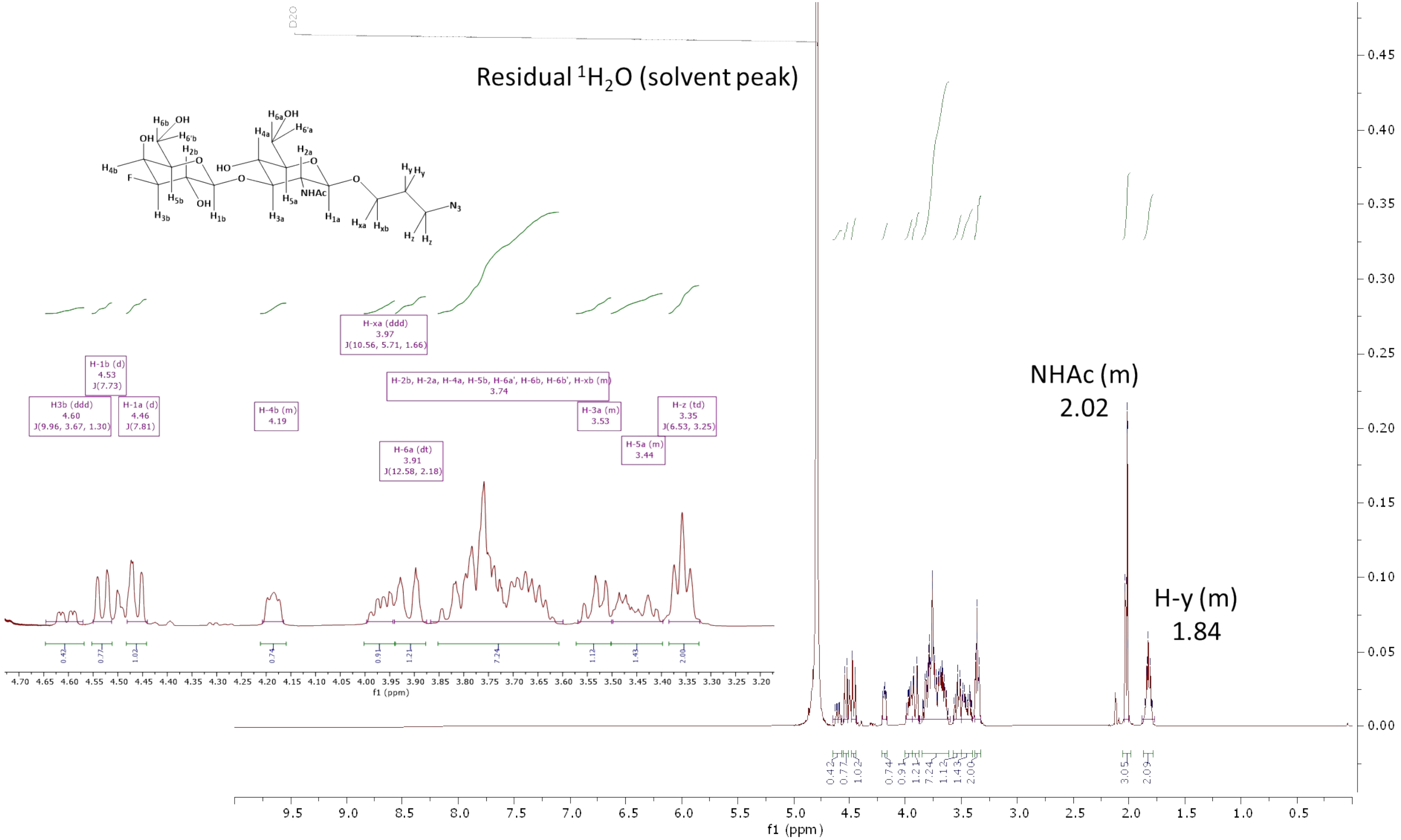

^{13}C NMR Spectrum for 3FGal-β(1,3)-GlcNAc-N_3

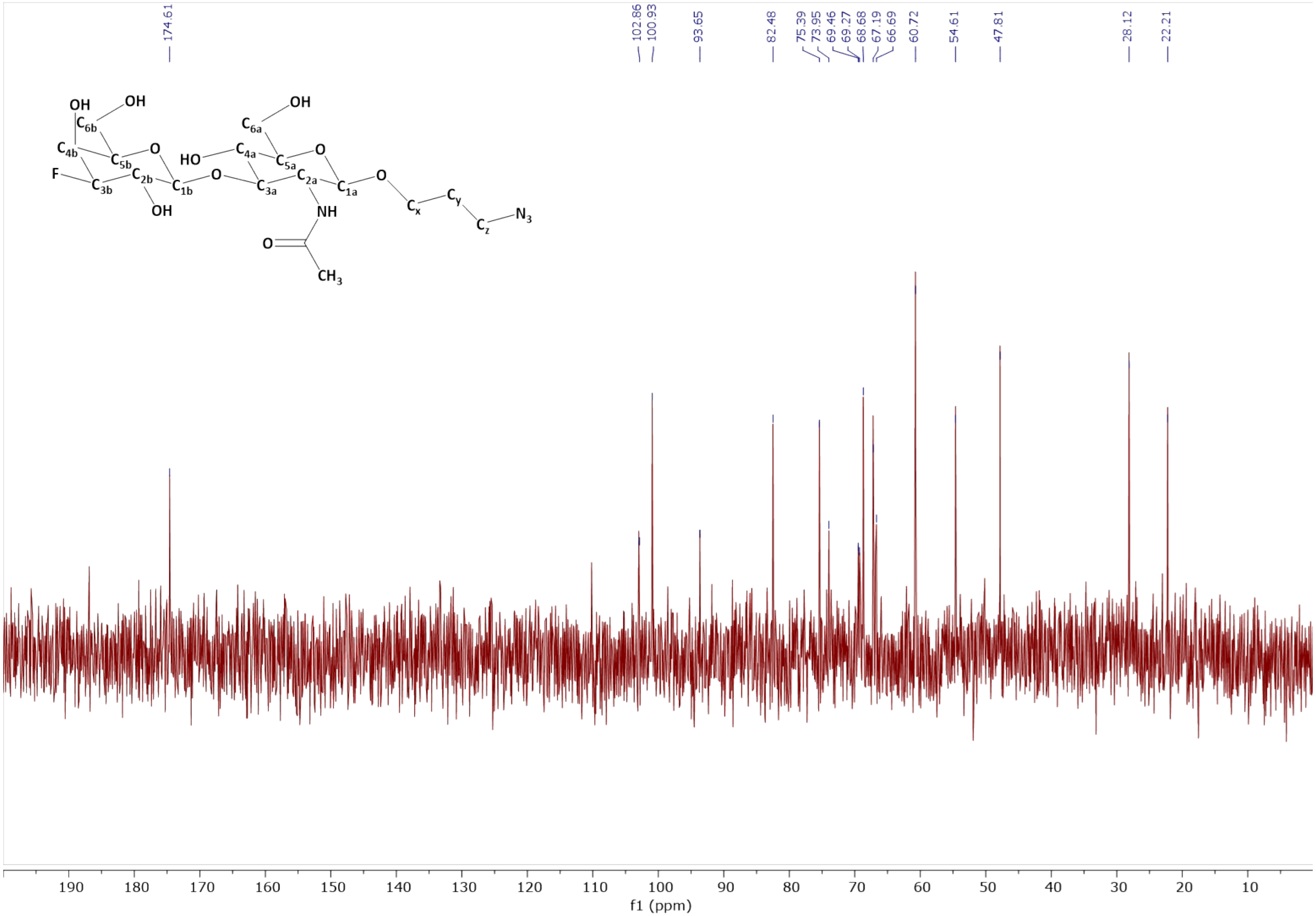

^{19}F NMR Spectrum for 3FGal-β(1,3)-GlcNAc-N_3

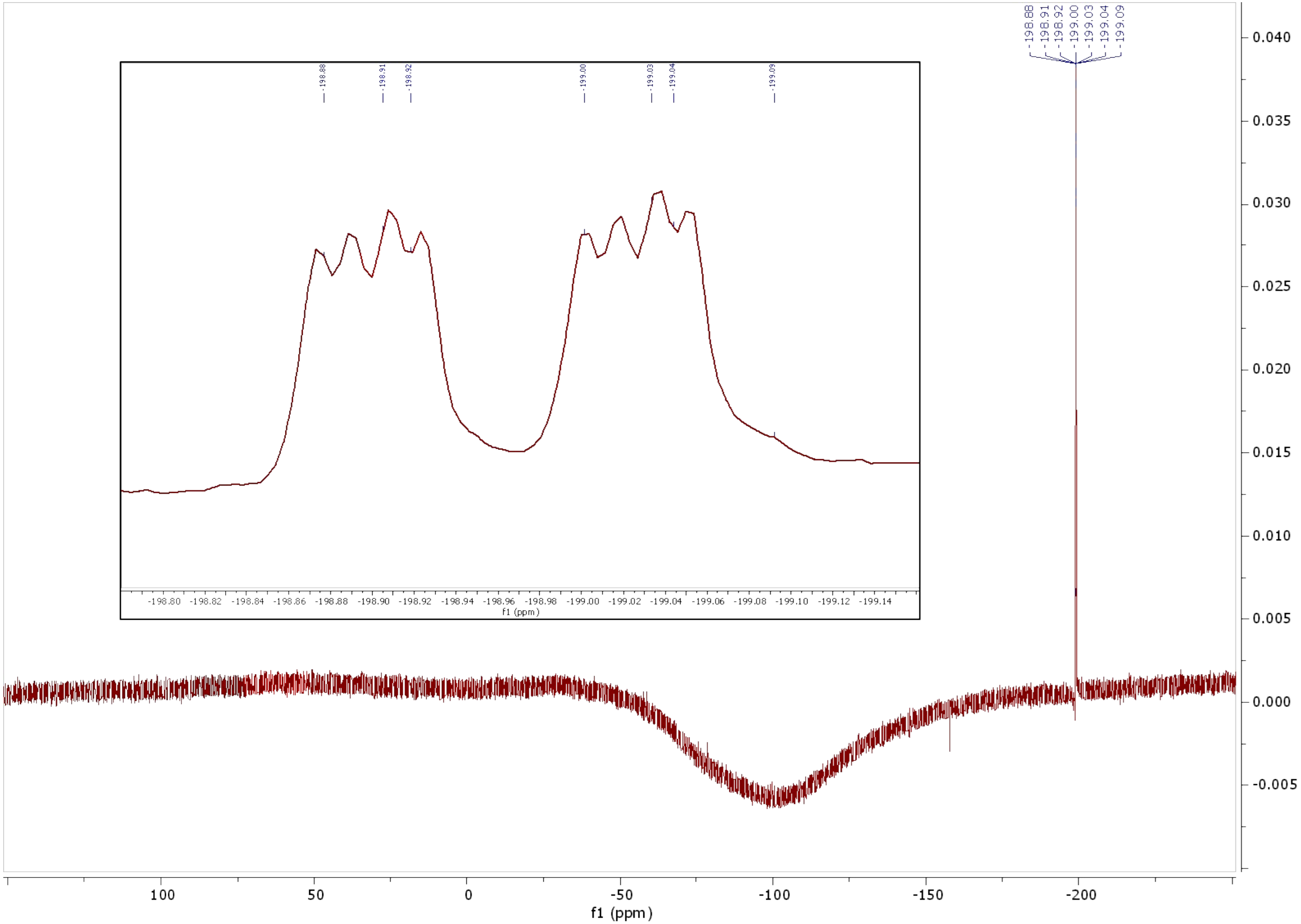

References

1 H. Brisset, A.-E. Navarro, F. Moggia, B. Jousselme, P. Blanchard and J. Roncali, *J. Electroanal. Chem.*, 2007, **603**, 149–154.

2 S. J. Richards, T. Keenan, J. B. Vendeville, D. E. Wheatley, H. Chidwick, D. Budhadev, C. E. Council, C. S. Webster, H. Ledru, A. N. Baker, M. Walker, M. C. Galan, B. Linclau, M. A. Fascione and M. I. Gibson, *Chem. Sci.*, 2021, **12**, 905–910.

3 Y. Xue, X. Li, H. Li and W. Zhang, *Nat. Commun.*, 2014, **5**, 1–9.

4 A. J. Bard and L. R. Faulkner, *Electrochemical Methods: Fundamentals and Applications*, John Wiley & Sons, Inc.., New York, NY, 2nd Edition., 2001.

5 M. F. Mousavi, M. Amiri, A. Noori and S. M. Khoshfetrat, *Electroanalysis*, 2017, **29**, 2818–2831.

6 M. A. Ali, K. Mondal, Y. Jiao, S. Oren, Z. Xu, A. Sharma and L. Dong, *ACS Appl. Mater. Interfaces*, 2016, **8**, 20570–20582.

7 S. M. Khoshfetrat, P. Seyed Dorraji, M. Shayan, F. Khatami and K. Omidfar, *Anal. Chem.*, 2022, **94**, 8005–8013.
